# Habitat Suitability and Environmental Niche Differentiation of Two Commercially Important *Solenocera* Shrimp Species in the Southern Yellow Sea and East China Sea (2018–2026)

**DOI:** 10.3390/biology15211950

**Published:** 2026-10-29

**Authors:** Min Xu, Hongmei Li, Yong Liu, Jiahao Wang, Lijian Xue, Jianzhong Ling, Huiyu Li

**Affiliations:** 1 Key Laboratory of Fisheries Remote Sensing Ministry of Agriculture and Rural Affairs, East China Sea Fisheries Research Institute, Chinese Academy of Fishery Sciences, Shanghai 200090, China; 2 Shanghai Society of Oceanology and Limnology, East China Sea Ecology Center, Ministry of Natural Resources, Shanghai 200062, China; 3 Atmosphere and Ocean Research Institute, The University of Tokyo, Chiba 277-8564, Japan; 4 School of Fisheries, Zhejiang Ocean University, Zhoushan 316022, China

**Keywords:** boosted regression tree, East China Sea, habitat suitability index, niche differentiation, *Solenocera crassicornis*, *Solenocera koelbeli*, Southern Yellow Sea, Tweedie generalized additive model

## Abstract

*Solenocera crassicornis* (H. Milne Edwards, 1837) and *Solenocera koelbeli* De Man, 1911 are two commercially important shrimp species in the coastal waters of China; however, their inter-annual variation, seasonal spatial habitat use, and environmental niche differentiation remain poorly understood. Based on fishery-independent bottom-trawl surveys conducted over 21 cruises from November 2018 to January 2026 in the Southern Yellow Sea and East China Sea, we integrated Tweedie generalized additive models (GAMs) and boosted regression trees (BRTs) to generate habitat suitability index (HSI) model outputs and examine the observed spatiotemporal patterns and environmental niches of the two species. Model outputs yielded hold-out AUC-ROC values > 0.75; the models showed moderate to good temporal generalization but weaker spatial generalization, particularly for *S. crassicornis*. Field observations showed that *S. crassicornis* is a nearshore species whose distribution is primarily driven by sea surface temperature, sea bottom temperature and sea bottom salinity; it exhibited seasonal distributional shifts, with higher occurrence at northern latitudes (32–35° N) in summer and more southerly distribution in winter. *S. koelbeli* was a deep-water, high-salinity species mainly associated with sea bottom salinity and depth, found at 50–100 m and virtually absent from low-salinity estuarine waters, with high occurrence in autumn–winter and late spring. Both shrimp species shared core habitats (Zhoushan, Yushan and Wentai) but displayed temporal, spatial, and environmental niche partitioning according to our observations and model outputs. These findings provide scientific references for resource conservation and fishing ground management. Potential seasonal protection windows for broodstocks (June–September) and recruitment stocks (December–February) are proposed based on field observations and correlative model outputs, although these require further empirical validation.

## Simple Summary

This study investigated habitat suitability and environmental niche differentiation for two commercially important *Solenocera* shrimps, *Solenocera crassicornis* and *S. koelbeli*, in the Southern Yellow Sea and East China Sea based on 21 fishery-independent bottom-trawl surveys. We found that *S. crassicornis* is a nearshore species with seasonal latitudinal shifts. In contrast, *S. koelbeli* is a deeper, high-salinity offshore species, one concentrated at depths of 50–100 m and rarely encountered in estuarine waters, with its peak occurrence in autumn–winter and late spring. The findings presented in this study provide scientific support for fishery conservation and ground management.

## 1. Introduction

Shrimps are currently the most economically valuable fishery product traded globally [1]. In many developing countries, such as China and India, they represent the most profitable fishery commodity [2,3]. The family Solenoceridae is of considerable economic importance to commercial fisheries [4]. Two commercially important *Solenocera* shrimp species are *Solenocera crassicornis* (H. Milne-Edwards, 1837) and *Solenocera koelbeli* De Man, 1911, which belong to the genus *Solenocera* Lucas, 1849, a genus comprising 38 recognized species [5].

*S. crassicornis* (Decapoda, Solenoceridae, *Solenocera*) is a medium-sized commercial shrimp with a body length of 50–100 mm [6]. This species has a one-year life span with body length and weight ranging from 50 to 90 mm and 5 to 9 g, respectively. It prefers muddy and sandy seabeds and mainly feeds on benthic organisms, small demersal nekton, and plankton [7]. *S. crassicornis* is distributed in India, Malaysia, Indonesia, the Arafura Sea, the Mergui Archipelago, Singapore, North Borneo, Japan, and China (including the Southern Yellow Sea, East China Sea, and South China Sea) [8]. Prior to the 1970s, *S. crassicornis* was mainly caught as bycatch by coastal stationary stake nets in China; after the 1980s, it has been targeted by beam shrimp trawlers operating north of 30° N in the East China Sea at depths < 60 m [6]. In the East China Sea, it ranks sixth among shrimp species after *Parapenaeus fissuroides*, *Metapenaeopsis longirostris*, *Palaemon gravieri*, *Metapenaeopsis barbata*, *and S. koelbeli* [6]. The annual catch in the East China Sea is approximately 10,000–20,000 tons; in Zhejiang, China, it accounts for 5.7% of the total shrimp catch [8]. Frozen peeled *S. crassicornis* products are an important marine commodity for export revenue [8].

*S. koelbeli* (Decapoda, Solenoceridae, *Solenocera*) is a medium-sized thermophilic and euryhaline shrimp species inhabiting warm, high-salinity waters and gravel, sandy, and muddy seabeds, with a one-year lifespan [9]. *S. koelbeli* feeds mainly on benthic invertebrates [10]. *S. koelbeli* is distributed in the Indo-West Pacific, including Japan, Malaysia, and China (the East China Sea and South China Sea) [11]. It occurs at depths of 60–120 m, with a salinity >33 and water temperatures of 12–24 °C; its preferred conditions are 33–34 with a sea bottom temperature of 20–24 °C [12]. The potential stock of *S. koelbeli* was estimated at 40,000 tons in Central and Southern Zhejiang waters and 1059 t in the Northern Taiwan Strait [10]. Little is known about the seasonal spatial distribution patterns as they relate to environmental factors for *S. crassicornis* and *S. koelbeli* in the Southern Yellow Sea and East China Sea.

In this study, based on standardized bottom-trawl survey data collected from the Southern Yellow Sea and East China Sea during 2018–2026, we integrated Tweedie generalized additive models and boosted regression trees to analyze habitat suitability, spatiotemporal patterns, and environmental niche differentiation of *S. crassicornis* and *S. koelbeli*. Second, we aimed to clarify the seasonal occurrence patterns, habitat preferences, and size-dependent differences in environmental adaptation of the two shrimp species, as well as to reveal inter-annual trends in their habitat suitability. The findings presented in this study can provide scientific references for resource conservation, fishing ground management, and sustainable fishery exploitation of the two commercially important *Solenocera* shrimp species in China’s coastal seas.

## 2. Materials and Methods

### 2.1. Field Survey and Data Collection

Standardized fishery-independent bottom trawl surveys targeting *S. crassicornis* and *S. koelbeli* were implemented across 21 sequential research cruises in the Southern Yellow Sea and East China Sea between 2018 and 2026. Information including the cruise identifier, survey period, season, total number of survey stations per cruise, and number of occurrence stations for *S. crassicornis* and *S. koelbeli* is summarized in Table 1. Sampling efforts varied substantially across cruises, seasons, and years. Some geographic areas were not covered by our surveys, and several cruises had reduced station numbers mainly due to COVID-19-related fieldwork restrictions. We mitigated this sampling imbalance primarily through model-based adjustments described above, although the residual bias cannot be fully eliminated (Table 1). No replicate trawl hauls were performed at individual stations; hence, pure sampling variance from a single trawl could not be fully eliminated from intrinsic biological variability.

A total of 21 survey cruises were conducted in this study, spanning November 2018 to January 2026 and covering all four seasons (spring, summer, autumn, and winter). The total number of survey stations per cruise ranged from 42 to 157 (Table 1). Overall, *S. crassicornis* occurred at more stations than *S. koelbeli*. *S. crassicornis* was not collected in May 2019; the number of stations with catches ranged from 4 to 38 for the remaining cruises. For *S. koelbeli*, the number of catch-positive stations varied from 0 to 26. High counts of stations with *S. crassicornis* catches were recorded in autumn, exceeding 35 stations in November 2018 and November 2021. In contrast, catch-positive stations of *S. koelbeli* in autumn showed marked inter-annual fluctuations. Both species showed a high occurrence in winter. In summer, the number of catch-positive stations for *S. crassicornis* generally ranged between 5 and 35, whereas *S. koelbeli* had fewer catch-positive stations in summer, with fewer than 10 stations in most cruises. In spring, both species had low numbers of catch-positive stations alongside substantial inter-annual variability (Table 1).

**Figure 1:**
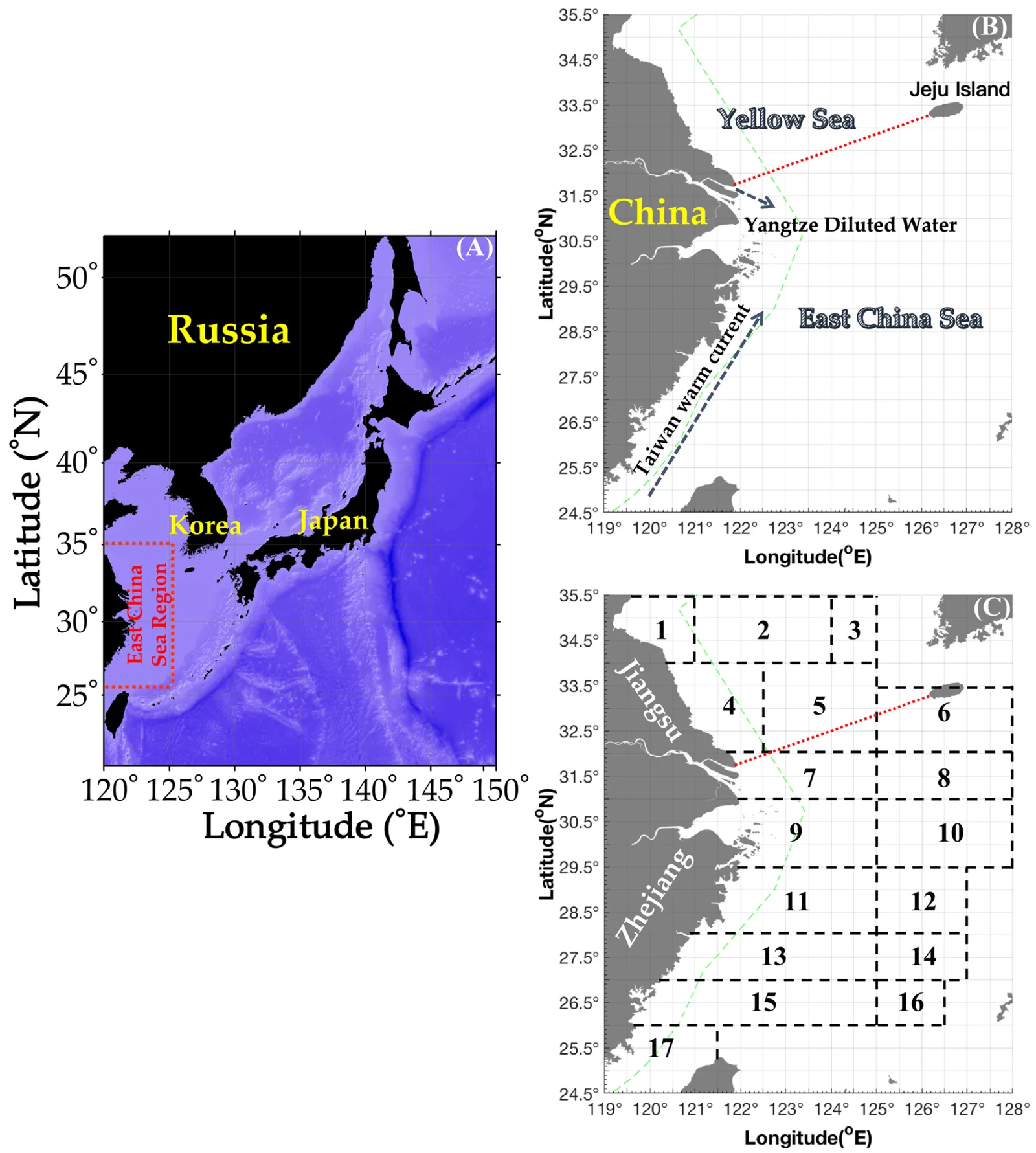
Schematic of the study area and surveyed fishing grounds. (**A**) Geographical scope of the study region (26.50–35.00° N, 120.00–127.00° E) (red dashed box). (**B**) Southern Yellow Sea and East China Sea, with their boundary marked by a red dashed line. The green dashed line marks the motor-trawl prohibition line. (**C**) Positions of 17 surveyed fishing grounds: (1) Haizhou Bay, (2) Lianqingshi, (3) Liandong, (4) Lvsi, (5) Dasha, (6) Shawai, (7) Yangtze River Estuary, (8) Jiangwai, (9) Zhoushan, (10) Zhouwai, (11) Yushan, (12) Yuwai, (13) Wentai, (14) Wenwai, (15) Mindong, (16) Minwai, and (17) Minzhong. The green dashed line marks the motor-trawl prohibition line. The red dashed line indicates the boundary between the Yellow and East China Seas.

Surveys were carried out aboard the fisheries research vessels Zhongkeyu 211 and Zhongkeyu 212 within 26.50–35.00° N and 120.00–127.00° E (Figure 1), with sampling stations deployed in a 30-arcminute-spaced grid (latitude/longitude). The standardized bottom trawl employed had a 20 mm cod-end mesh with a 102-mesh opening, 72.24 m headline length, 10–15 m net height, and 82.44 m groundline length. Each station involved one hour of trawling at a constant speed of 3–4 knots. Only a single trawl haul was completed at each sampling station. All specimens collected were immediately transported to the laboratory post-trawling for species identification and to record their presence at each sampling station. At each station, the entire catch was enumerated, and the total wet weight was determined with a precision of 0.10 g. Two CPUE indices were derived for the target species: biomass density (CPUE_w_, g·h^−1^) and numerical density (CPUE_n_, ind·h^−1^). Catch per unit effort (CPUE) was computed according to the following equations of CPUE_w_ = *W_i_*_/_*t_i_* and CPUE_n_ = *N_i_*_/_*t_i_*, where *N_i_* is the number of individuals caught at station *i* (ind), *W_i_* is the catch weight at station *i* (g), and *t_i_* is the trawling duration at station *i* (h). Notably, CPUE_w_ and CPUE_n_ in this study serve as relative-density proxies rather than absolute abundance estimates; they are affected by variability in trawl catchability and gear selectivity. The average individual weight (AIW) per station was then estimated as the ratio of CPUE_w_ to CPUE_n_. We measured the body-length samples of *S. crassicornis* collected in August 2019, November 2021, September 2023, December 2023, and January 2025, as well as the body-length samples of *S. koelbeli* collected in November 2018, January 2019, May 2019, September 2019, April 2024, May 2025, and November 2025.

Water depth, temperature, and salinity were measured at each sampling station via a conductivity–temperature–depth (CTD) profiler (SBE 19, Sea-Bird Scientific, Bellevue, WA, USA). Sea surface salinity (SSS) and sea surface temperatures (SSTs) were recorded 3 m below the sea surface, whereas bottom salinity (SBS) and bottom temperatures (SBTs) were sampled at 2 m above the seafloor (for depths < 50 m) and 2–4 m (for depths > 50 m) above the seafloor.

### 2.2. Habitat Suitability Modeling Using Tweedie GAMs and Boosted Regression Trees

We selected two shrimp species of the genus *Solenocera* as the study species. The compiled dataset included spatial coordinates (longitude and latitude), temporal information (year and season), abundance records, and environmental variables, including: SST, SSS, SBT, SBS, and water depth. Only two sampling stations from the January 2019 cruise had missing environmental data. These records were excluded before running the analytical scripts, leaving 109 valid station records for the two *Solenocera* species in January 2019. Year and season were treated as categorical variables to build a standardized dataset for subsequent analyses.

Tweedie generalized additive models (GAMs) were fitted to zero-inflated numerical CPUE data to develop univariate environmental suitability indices (SIs) for each hydrological predictor. A Tweedie distribution with a natural logarithm link function was adopted for GAM fitting to accommodate zero-enriched fishery catch data, obviating separate zero-inflation stratification with a hurdle model. The model form was specified as follows:

*g*(*µ*) ~ *s*(*env_var*, *k* = 4, *bs* = ‘*cr*’) + *s*(*lon*, *lat*, *bs* = ‘*tp*’) + *season* + *s*(*year*, *bs* = ‘*re*’) (1)

where *µ* represents the station-specific expected numerical CPUE. The one-dimensional penalized cubic regression spline *s*(*env_var*, *k* = 4, *bs* = ‘*cr*’) was specified for each environmental covariate; notably, if an environmental variable had fewer than four unique values, the smooth term was simplified to a linear term to maintain model stability. The basis dimension *k* = 4 was selected for each environmental cubic regression spline to balance model flexibility and sample size; the adequacy of *k* was checked using gam.check() diagnostics from the mgcv package. The two-dimensional thin-plate spline term *s*(*lon*, *lat*, *bs* = ‘*tp*’) was included for spatial smoothing to account for spatial autocorrelation between sampling stations. Season was fitted as a categorical fixed effect to capture seasonal variations in abundance. The random smooth *s*(*year*, *bs* = ‘*re*’) accounts for inter-annual variability and mitigates partial confounding between uneven seasonal sampling and inherent seasonal population cycles. This term separates year-specific noise from species–environment relationships instead of inferring long-term population trends. We recognize that limited year levels may increase uncertainty of this term, and temporal cross-validation was implemented to evaluate model robustness.

Spatial cross-validation was implemented using blockCV spatially blocked partitioning when available; otherwise, *k*-means clustering on station longitude and latitude was used to create 20 spatial groups. We note that *k*-means clusters are not always spatially contiguous, and samples from the same cruise or repeated samples at identical coordinates may be allocated to different folds. For AUC-ROC calculations, continuous CPUE values were converted into binary presence–absence records (1 for CPUE > 0, 0 for CPUE = 0). The cleaned dataset was partitioned into 70% training and 30% independent hold-out subsets via stratified random sampling stratified by year and species presence/absence, with a fixed random seed for reproducibility.

Concurvity, representing multicollinearity among penalized smooth terms, was calculated using the concurvity() function from the R mgcv package with the argument full = TRUE. Maximum and mean concurvity statistics were extracted for each smooth term of environmental covariates. A threshold of 0.8 was applied to identify severe concurvity; predictors exceeding this cutoff were regarded as having a high multicollinearity risk. Excessively high concurvity may produce unstable model estimates and underestimated standard errors. The worst-case concurvity describes concurvity under the worst-case scenario, representing the maximum proportion to which an environmental variable can be explained by a linear combination of all other predictors (primarily longitude, latitude and year). The observed-case concurvity is calculated from empirical data and reflects the average degree of dependence in the real dataset. The estimated-case concurvity refers to the estimated concurvity value.

Despite the prevalence of zero catches in the dataset, a two-stage hurdle model comprising distinct zero- and count-component sub-models was not implemented. Instead, the Tweedie compound Poisson distribution was employed to jointly fit zero-enriched catch observations, eliminating the need for separate zero-inflation stratification. Based on model-derived predictions, min–max normalization was used to rescale univariate response values to the range 0–1, producing the suitability index (SI) for each environmental predictor. An SI approaching 1 denotes more favorable environmental conditions for the distribution of the target species. During SI normalization, predicted suitability values were bound to the biologically plausible range [0, 1]. The associated 95% confidence intervals were concurrently truncated between 0 and 1 to prevent generation of ecologically implausible out-of-bounds suitability estimates.

Boosted regression tree (BRT) models were implemented using the gbm.step function in the dismo package to quantify variable relative importance for subsequent HSI weighting. Considering the data distribution characteristics of discrete zero-inflated CPUE records, the BRT model preferentially adopted a Poisson distribution for nonnegative integer CPUE data, with a Gaussian distribution as an alternative if Poisson fitting failed. BRT hyperparameters were uniformly set as follows: tree complexity = 20, Poisson learning rate = 0.005, Gaussian learning rate = 0.01, and a bag fraction of 0.6. Tree complexity represented the complexity of individual regression trees. Each tree was allowed to have a maximum depth of 20 splits and up to 20th-order variable interactions. This configuration enabled the model to capture complex synergistic effects among multiple environmental factors, which was suitable for marine systems where biological distributions were driven by coupled multi-factor influences. A smaller learning rate reduced the contribution of each individual tree; consequently, more trees were required for model convergence, which improved generalization and mitigated overfitting. The bag fraction was set to 0.6. In each training iteration, 60% of samples were randomly drawn to fit a single tree (sub-sampling). Ranging from 0 to 1, this parameter acted as a regularizer via random sub-sampling, reduced correlation among individual trees and suppressed overfitting (Table S1).

A 20-fold cross-validation framework was applied to automatically identify the optimal number of trees with a maximum tree limit of 5000 instead of presetting a fixed tree number. To avoid cross-validation information leakage, BRT variable importance weights were refitted independently within each temporal and spatial cross-validation fold. The gbm.step function automatically produced three key outputs: the optimal total number of trees associated with minimum cross-validation deviance; cross-validation correlation coefficient, training and cross-validation deviance, together with mean squared error (MSE); and raw relative variable influence values (*rel.inf*). *Rel.inf* refers to the relative influence output by the BRT model, which reflects the overall contribution of variables to ensemble prediction; higher values indicate greater involvement of the environmental factor in constructing prediction splits for target-species stock abundance. Subsequent post-processing kept only the top-five dominant environmental predictors and performed secondary normalization:

(2) $$W_{i}=\frac{\mathrm{rel}.\inf_{i}}{\sum \mathrm{rel}.\inf_{\mathrm{env}}}$$

Using the Arithmetic Mean Model (AMM), the composite habitat suitability index (HSI) was computed as the weighted sum of single-factor suitability index (SI) values and their corresponding variable weights as HSI = $\sum_{i=1}^{n}\left({\mathrm{SI}}_{i}\times {\mathrm{Weight}}_{i}\right)$, where SI_i_ is the suitability index for the i-th environmental factor, Weight_i_ represents its associated weight, and n refers to the total number of environmental variables. For each environmental covariate, normalized weights summing to one were generated. These weights were then used to weight and aggregate single-factor suitability index (SI) values to compute the composite habitat suitability index (HSI). Mathematically, these normalized weights are derived from the relative influence metric of the BRT ensemble and are model-configuration dependent; they do not quantify independently partitioned deviance for each predictor. Ecologically, these weights are model-specific outputs rather than universal ecological weights; alternative model configurations may yield different weighting results.

The hold-out number of test observations represented the independent test dataset. Temporal cross-validation generated mean and SD metrics based on temporal partitioning, which reflected the temporal stability of the model. Spatial cross-validation, for which training and test sets were partitioned using the k-means spatial cluster algorithm, yielded mean and SD metrics based on spatial partitioning and assessed the model’s generalization across different geographic locations. RMSE (root-mean-square error) quantified the average magnitude of discrepancy between predicted and observed values (Table S1). AUC-ROCs measured the model’s capacity to discriminate presence–absence (e.g., occurrence versus non-occurrence in species distribution studies). An AUC-ROC > 0.8 was generally regarded as indicative of good discriminatory power. Cross-validation folds denoted the number of cross-validation partitions. Deviance explained represented the proportion of data variance accounted for by the model.

Uncertainty for outputs from the generalized additive model (GAM) was quantified using 95% parametric confidence bands surrounding the SI response curves, which represented uncertainty in suitability responses along individual environmental gradients. For the boosted regression tree (BRT) model, 20-fold cross-validation was implemented to reduce overfitting, and cross-validation error characterized the overall predictive uncertainty of the BRT framework. Univariate suitability indices were min–max normalized using the full training dataset and combined via weighted arithmetic averaging. CPUE was interpreted as a relative proxy for habitat suitability rather than survival. Spatial and temporal cross-validation used data partitions independent from model training. Composite HSI outputs were further classified into three discrete habitat-quality tiers: highly suitable habitats (HSI ≥ 0.7), moderately suitable habitats (0.4 ≤ HSI < 0.7), and unsuitable habitats (HSI < 0.4). The resulting HSI should be interpreted as a relative data-driven index for comparing spatiotemporal suitability patterns within the study area and not an absolute measure of habitat quality. All data preprocessing, model development, index calculation, statistical analyses, and visualizations were completed using R software (version 4.5.2).

## 3. Results

### 3.1. Model Performance and Validation of Habitat Suitability for S. crassicornis and S. koelbeli

Model cross-validation produced hold-out AUC-ROC values greater than 0.75. Instead of qualitative labels, such as “moderate to good”, numerical cross-validation metrics indicated that models attained acceptable predictive performance, with stronger temporal (*S. crassicornis*: 0.7565 ± 0.0958; *S. koelbeli*: 0.8789 ± 0.0324, Table S1) than spatial generalization (*S. crassicornis*: 0.5921 ± 0.1652; *S. koelbeli*: 0.6901 ± 0.2054, Table S1). We noted that high AUC-ROC values may partly reflect the underlying spatial sampling structure captured by the two-dimensional spatial spline term rather than just species–environment niche responses. Temporal cross-validation yielded high AUC-ROCs with small standard deviations, indicating stable predictive performance over the years, while numerical prediction errors remained relatively low for both species (Table S1). For *S. crassicornis*, SST, SBT, and SBS together dominated the BRT relative influence (cumulative ~60%). For *S. koelbeli*, SBS and depth dominated the relative influence (cumulative ~50%), highlighting its strong dependence on benthic habitats; SST and SSS exerted only minor influences. Concurvity was low for both species (Table S2).

Annual temporal cross-validation produced mostly AUC-ROC values above 0.8 across the 8-year study period, demonstrating good temporal generalization (*S. crassicornis*: 0.7232–0.9107; *S. koelbeli*: 0.843–0.9273, Table S3). Residual diagnostics supported the suitability of the Tweedie distribution for our zero-inflated fishery catch data. No systematic directional bias was evident in model predictions, and structured variation was adequately captured by spatial thin-plate splines, year random smooths, and seasonal fixed effects. Cross-validation, BRT-based weighting, and bootstrap uncertainty evaluation reduced prediction errors originating from extreme observations. Observed species occurrences generally aligned with higher HSI values (Figure S1). The correspondence between species occurrence and HSI bins served only as an internal sanity check of model outputs and did not represent independent external validation. The model’s predictive performance was primarily assessed using spatial and temporal cross-validation.

Kernel-density plots of HSIs revealed niche differentiation between the two shrimp species (Figure S2). *S. crassicornis* exhibited substantial overlap between presence and absence records at moderate HSIs (0.4–0.7), reflecting a broader habitat niche breadth. In contrast, *S. koelbeli* presence records clustered at very high HSIs (>0.7), indicating more stringent habitat requirements.

### 3.2. Analyses of Body-Length Frequency, Growth Traits, and Population Ecology of Two Solenocera Species

Most specimens of *S. crassicornis* had body lengths ranging from 45 mm to 75 mm. Individuals exceeding 105 mm in body length were collected at Zhoushan in August 2019, where females outnumbered males, with a mean body length of 73.6 mm. In January 2025, the mean body lengths were 55.3 mm and 61.1 mm at Shawai and Jiangwai, respectively, with equal numbers of females and males. This suggests that smaller-sized individuals aggregate in offshore waters of the Southern Yellow Sea and the Yangtze River Estuary during winter, and these areas are hypothesized to be potential overwintering grounds for juveniles. In terms of sexual maturity, a considerable number of individuals at maturity stages IV and V occurred in Zhoushan waters in summer, leading to the hypothesis that the Zhoushan fishing ground may serve as its potential spawning ground (Figure S3).

Body-length frequency distributions revealed that *S. koelbeli* attains distinctly larger body sizes than *S. crassicornis*, with most individuals falling within 65–95 mm. The mean body lengths exceeded 80 mm at Wentai and Mindong fishing grounds in May, and individuals larger than 105 mm were recorded at Wentai. The mean body length at Zhoushan in January was relatively small, at only 76.6 mm. The sex ratio fluctuated considerably across months. It is hypothesized that females and males may arrive at the spawning ground in separate batches. In terms of gonadal maturity, numerous individuals were either immature or undergoing gonadal development at Wentai in April, whereas sexually mature individuals had already been recorded at the Mindong fishing ground in May. Furthermore, shifts in the dominant body-length group were observed at the Yushan fishing ground in November 2018 and November 2025, indicating inter-annual differences in population growth and recruitment (Figure S3).

**Figure 2:**
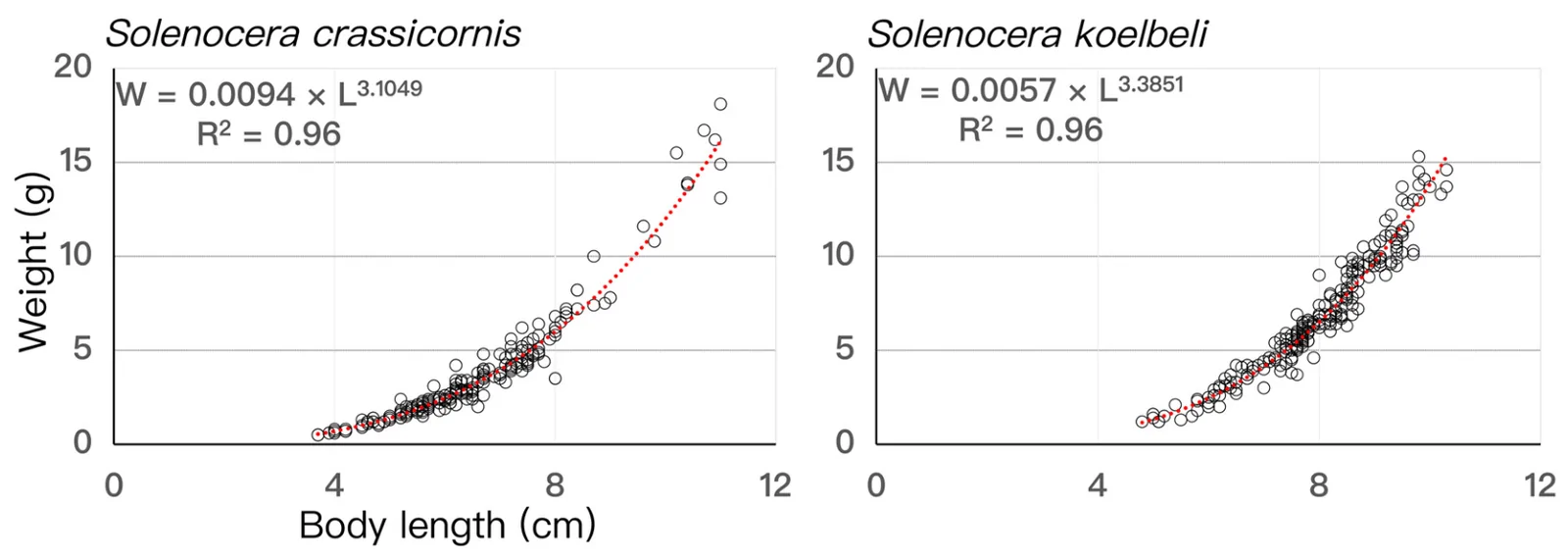
Length–weight fitted relationships for *Solenocera crassicornis* and *Solenocera koelbeli*.

Scatter-plot trends showed strong consistency between the two species. At small body lengths (0–4 cm), body weight remained low with limited dispersion. Once body length exceeded 4 cm, body weight increased sharply, accompanied by elevated dispersion in individual weight, reflecting greater variability in body condition among individuals of the same body length or age. The higher *b* value (3.3851 > 3.1049) indicated a stronger positive allometric length–weight relationship in *S. koelbeli* (Figure 2).

### 3.3. Temporal Patterns of CPUE_w_, CPUE_n_, and AIW of Two Solenocera Shrimp Species

‘Total CPUE’ in this study refers to the sum of station-level CPUE values across all valid sampling stations within a given survey cruise; it represents aggregate catch intensity over sampled stations and is not an estimate of absolute population abundance for the entire study area. The total CPUE_w_ of *S. crassicornis* peaked in November 2021 (autumn), followed by a secondary peak in August 2020 (summer). High total CPUE_w_ mainly occurred in summer (August–September), whereas CPUE_w_ was generally below 4000 g·h^−1^ in spring and winter (January, May, and December). Spring (May) consistently maintained low values ranging from 355 to 1924 g·h^−1^. In terms of inter-annual variation, total CPUE_w_ of *S. crassicornis* remained <10,000 g·h^−1^ from 2018 to 2019. It surged above 20,000 g·h^−1^ in August 2020 and November 2021, yet it reached historical minima in May 2020 and May 2021 before declining back to levels < 10,000 g·h^−1^ from 2022 to 2025 (Table 2).

The total CPUE_w_ of *S. koelbeli* reached its maximum in November 2021 (autumn). In terms of inter-annual variation, total CPUE_w_ in winter exceeded 10,000 g·h^−1^ during 2018 to 2019, whereas values were low in spring and summer. From 2020 to 2023, values stayed low in most months except for the anomalous high catch recorded in November 2021. During 2024 to 2025, spring values rebounded to >10,000 g·h^−1^. As indicated by total CPUE_w_ and total CPUE_n_, *S. crassicornis* dominated in summer, whereas *S. koelbeli* prevailed in autumn–winter, with its peak CPUE reaching three times that of *S. crassicornis* (Table 2).

**Figure 3:**
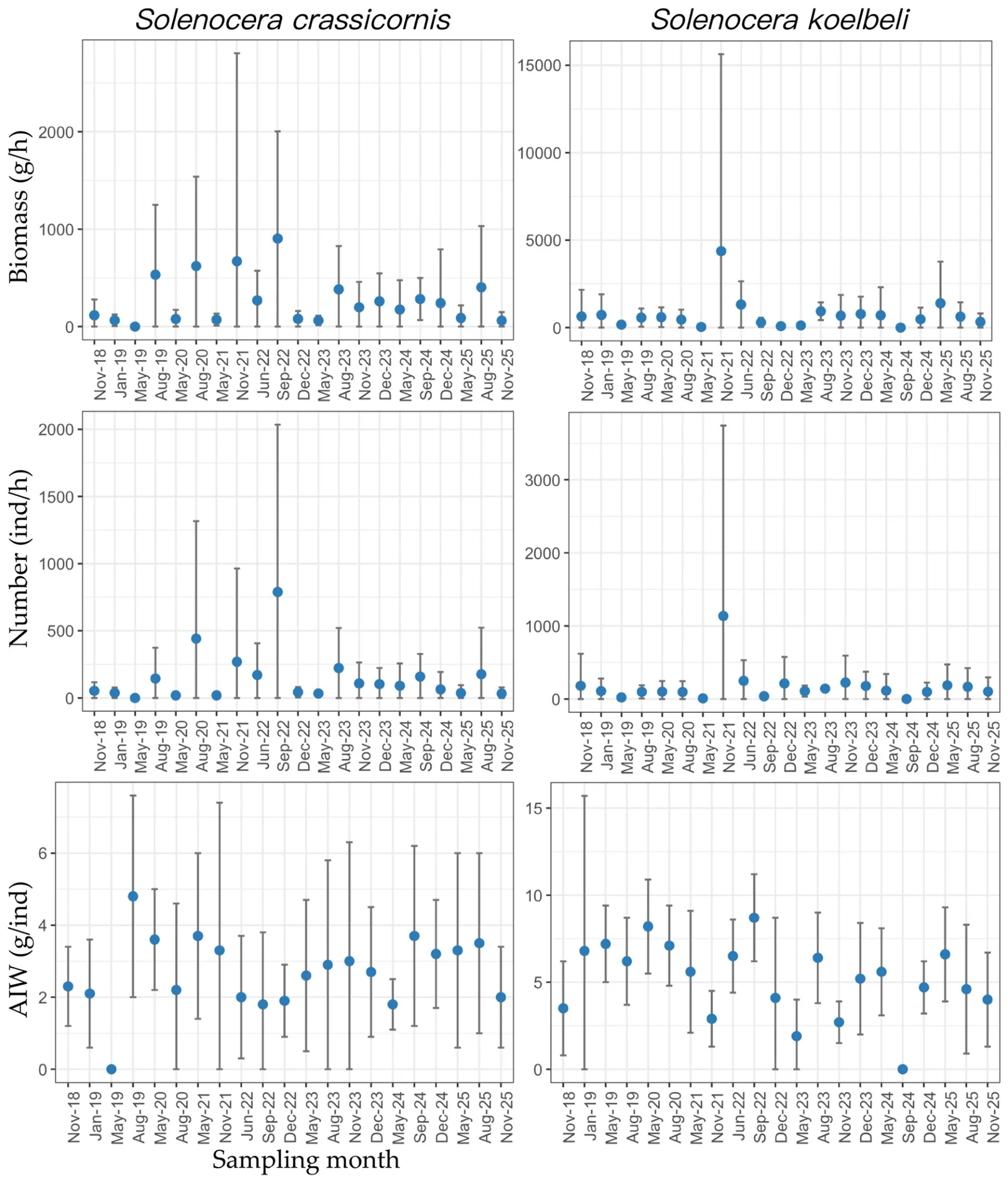
Temporal variations in CPUE_w_ (biomass; g·h^−1^), CPUE_n_ (number; ind·h^−1^), and average individual weight (AIW; g·ind^−1^) of *Solenocera crassicornis* and *S. koelbeli.* Blue dots represent the mean values across sampling stations, and error bars indicate standard deviation. No catch of *S. crassicornis* was recorded in May 2019, and no catches of *S. koelbeli* in September 2024.

Mean CPUE_w_ and CPUE_n_ of *S. crassicornis* peaked in September 2022, while the mean AIW was only 1.8 g·ind^−1^. Secondary peaks were observed in August 2020 and November 2021, and these periods were also characterized by high abundance of individuals with low AIW values. Our data showed that August, September, and November generally corresponded to periods of high abundance for this species. In terms of mean AIWs, August 2019 yielded the maximum value (4.8 g·ind^−1^) among all survey months, followed by May 2020, May 2021, and September 2024. Mean AIW values were approximately 2 g·ind^−1^ or lower in June 2022, September 2022, December 2022, and May 2024. *S. crassicornis* exhibited relatively high overall stock levels from 2018 to 2020. Extremely high abundance occurred at the Yushan fishing ground in autumn 2021. CPUE_w_ remained high across multiple summer–autumn survey cruises from 2022 to 2023 (Figure 3 and Table S4).

A high mean biomass and abundance of *S. koelbeli* were observed in November 2021, yet the mean AIW was only 2.9 g·ind^−1^. In May 2021, the mean biomass and abundance both reached their minima, with a mean AIW of 5.6 g·ind^−1^. May (spring) exhibited substantial inter-annual variability. In August (summer), the mean CPUE_w_ across years ranged from 456.3 to 935.5 g·h^−1^, with a mean AIW of around 6 g·ind^−1^. The maximum mean AIW across all survey months (8.7 g·ind^−1^) was recorded in September 2022; nevertheless, the mean CPUE_w_ for this month was only 297.7 g·h^−1^. In November (autumn), excluding the anomalous high-catch event in 2021, mean CPUE_w_ ranged from 300 to 700 g·h^−1^. The CPUE_w_ of *S. koelbeli* increased continuously from 2018 to 2021 and reached its maximum value in the dataset in November 2021. The Yushan–Wentai area remained the core distribution area from 2022 to 2025 (Figure 3 and Table S4).

### 3.4. Spatial Patterns of CPUE_w_, CPUE_n_, and AIW of Two Solenocera Shrimp Species

*Solenocera crassicornis* was mainly distributed in the coastal waters of the East China Sea, including Zhoushan, Yushan, and Wentai, while Dasha, Yangtze River Estuary, and Jiangwai fishing grounds constituted its secondary distribution areas. Specimens were collected at Lvsi only in November 2018, and sparse occurrences were recorded at Mindong only in January and August 2019. Its frequent presence in the Yangtze River Estuary and Dasha fishing grounds reflects its association with areas subject to large salinity fluctuations driven by estuarine diluted water. High CPUE_w_ values ranging from the hundreds to one thousand were observed in multiple cruises during August–September (summer). CPUE_w_ further decreased in winter (December–January), whereas its proportion increased in the northern survey area (Dasha, Yangtze River Estuary, Jiangwai), suggesting a population shift toward the northwest coastal zone. AIW in winter was generally 1–3 g·ind^−1^. Within a single survey cruise, AIW values at Yushan and Wentai were frequently higher than those at Dasha and the Yangtze River Estuary. Ranked by the maximum CPUE_w_ at each fishing ground, the order for *S. crassicornis* was Yushan (2787.4 g·h^−1^) > Zhoushan (1540.2 g·h^−1^) > Wentai (1184.2 g·h^−1^) > Dasha (632.5 g·h^−1^) > Yangtze River Estuary (518.9 g·h^−1^) (Table S4).

**Figure 4:**
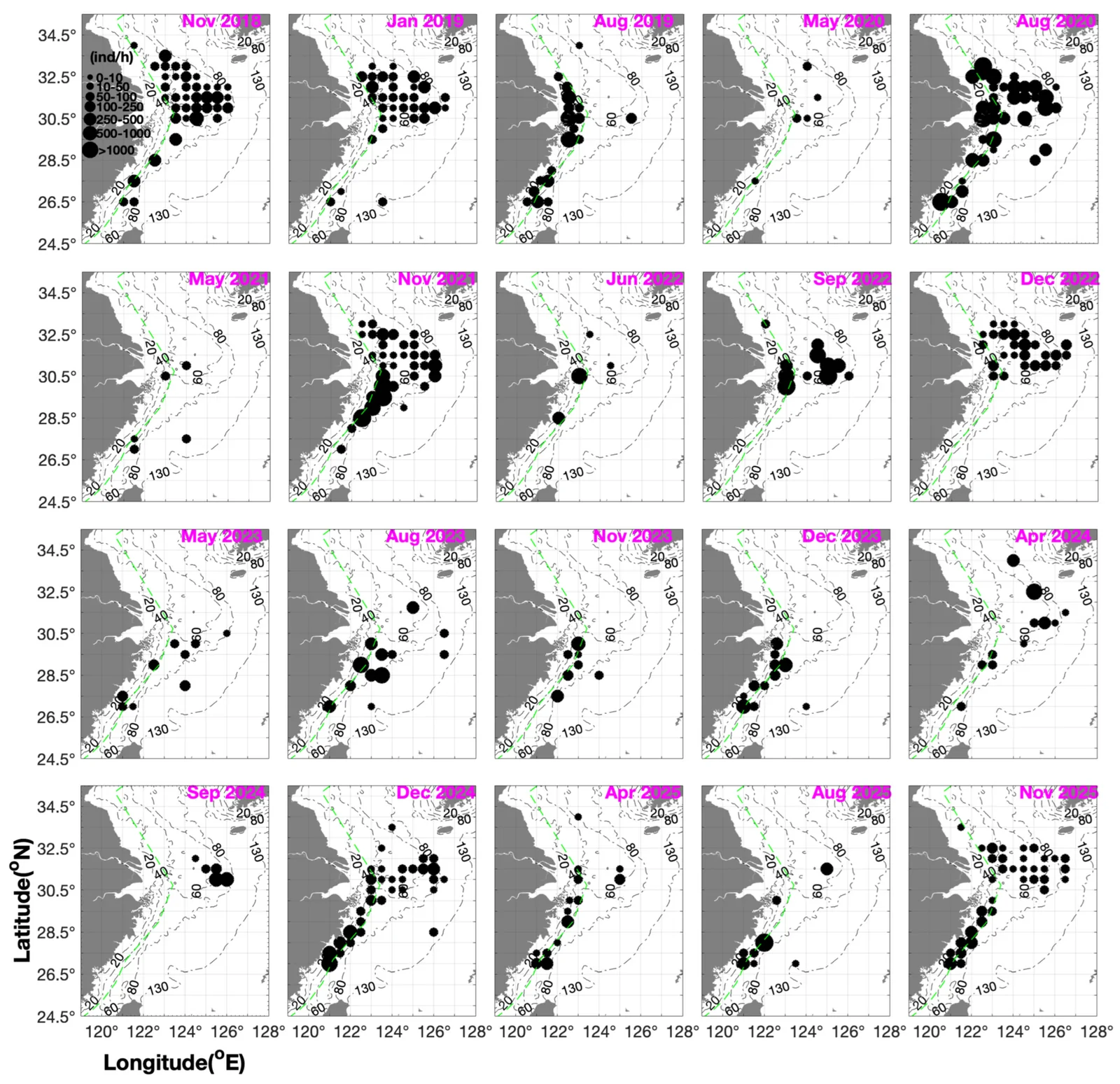
Monthly spatial distribution of CPUE_n_ for *Solenocera crassicornis* in the Southern Yellow Sea and East China Sea, spanning 2018 to 2025. No catch of *S. crassicornis* was recorded in May 2019. The green dashed line marks the motor-trawl prohibition line. Black circle size indicates CPUE_n_ (ind/h) at sampling stations.

According to model-derived HSI outputs, the core habitat lay in the Zhoushan–Yushan sea area (28–31° N, 122–124° E), and the adjacent waters of the Yangtze River Estuary formed seasonally suitable habitats in summer and autumn. The extent of model-derived suitable habitats showed a declining trend from 2018 to 2025 (Figure 4 and Figure S4).

For *S. koelbeli*, Yushan and Wentai represented the dominant distributional waters. No individuals were found in the Southern Yellow Sea, including the Dasha, Yangtze River Estuary, and Lvsi fishing grounds. This species was observed to occur in deeper waters, with most survey records occurring at depths of 50–100 m, and it was virtually absent from estuarine low-salinity waters. The AIW ranged from 0.9 g·ind^−1^ to 10.3 g·ind^−1^, with an overall mean AIW higher than that of *S. crassicornis;* higher AIW values occurred in spring and summer. The ranking of *S. koelbeli* was Wentai (12,050.3 g·h^−1^) > Yushan (3871.2 g·h^−1^) > Zhouwai (1276.5 g·h^−1^) > Yuwai (695.8 g·h^−1^) > Mindong (328.3 g·h^−1^). Although the spatial distribution range of *S. koelbeli* was narrower than that of *S. crassicornis*, it exhibited higher abundance and density within its occupied areas (Table S4).

**Figure 5:**
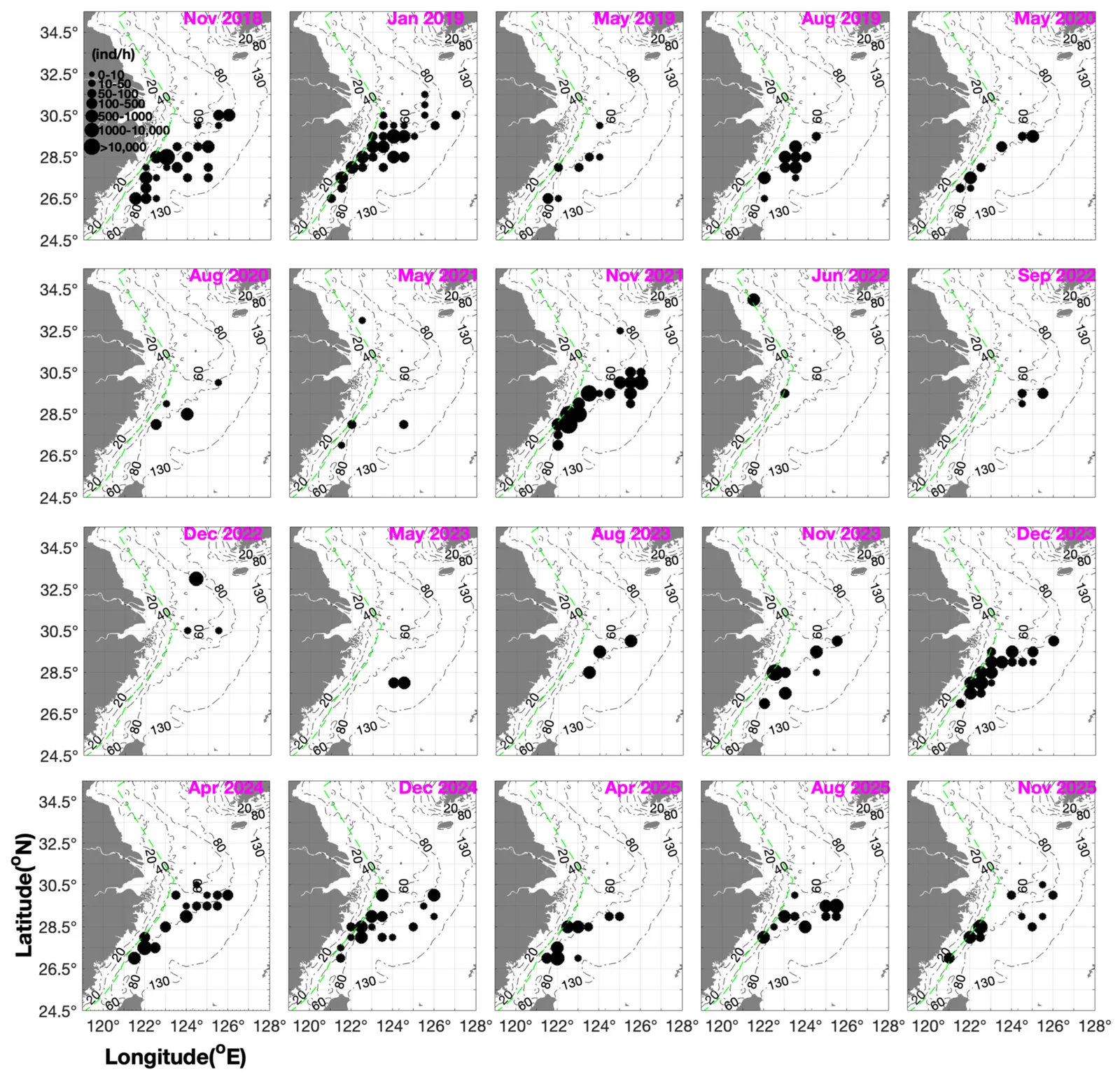
Monthly spatial distribution of CPUE_n_ for *Solenocera koelbeli* in the Ssouthern Yellow Sea and East China Sea, spanning 2018 to 2025. No catch of *S. koelbeli* in September 2024. The green dashed line marks the motor-trawl prohibition line. Black circle size indicates CPUE_n_ (ind/h) at sampling stations.

The model-derived highly suitable habitats of *S. koelbeli* were located further south. In spring, prominent highly suitable areas emerged in the Yushan–Wentai waters (27–29.5° N). The core fishing grounds lay in the offshore waters off Zhejiang, and almost no highly suitable habitats occurred north of 32° N. Autumn (November) was the season with the greatest extent of highly suitable habitats for *S. koelbeli* (Figure 5 and Figure S4).

### 3.5. Responses of Catch Metrics to Environmental Conditions for Solenocera crassicornis and S. koelbeli: Seasonal and Body-Size-Dependent Niche Patterns

**Figure 6:**
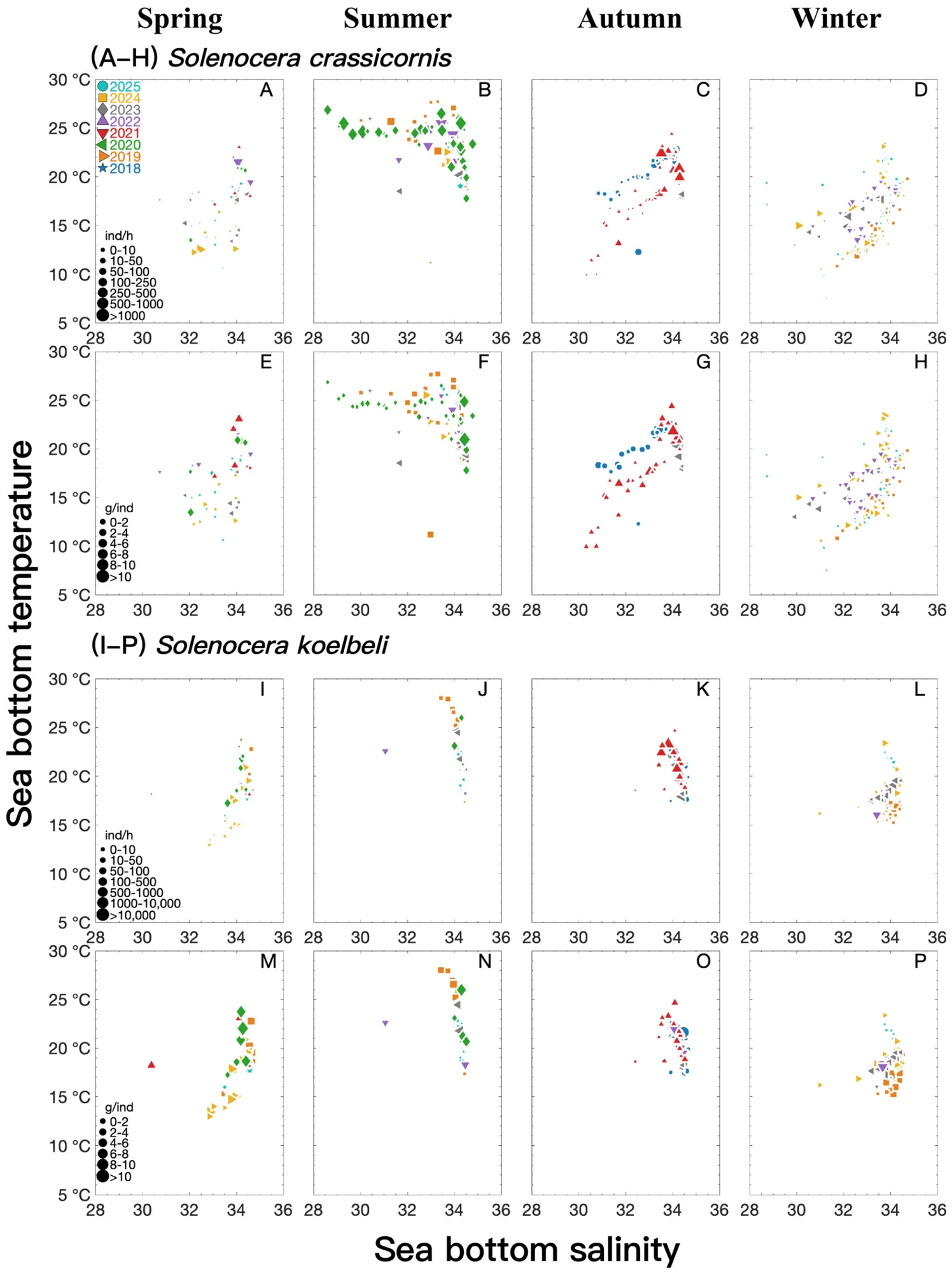
Relationships between sea bottom salinity, sea bottom temperature (°C), and two biological metrics (CPUE_n_ and AIW) of *Solenocera crassicornis* (**A**–**H**) and *Solenocera koelbeli* (**I**–**P**) across the seasons (spring, summer, autumn, and winter). Symbol sizes represent CPUE_n_ (ind·h^−1^; (**A**–**D**,**I**–**L**)) and average individual weight (g·ind^−1^; (**E**–**H**,**M**–**P**)), respectively. Different symbols and colors represent sampling survey years from 2018 to 2025: blue stars for 2018, orange right-pointing triangles for 2019, green left-pointing triangles for 2020, red down-pointing triangles for 2021, purple up-pointing triangles for 2022, gray diamonds for 2023, golden squares for 2024, and cyan circles for 2025.

*Solenocera crassicornis* occurred over a depth range of 7–145 m and was frequently found in waters shallower than 40 m. In spring (April–June), SSTs ranged from 14 °C to 25 °C. Even when thermal conditions were favorable, CPUE remained low in most fishing grounds. High CPUE (including CPUE_w_ and CPUE_n_) was more likely to occur when SBT > 19 °C in summer, whereas CPUE decreased markedly when SBT < 18 °C. Records associated with a low mean AIW (<1 g·ind^−1^) occurred across a wide salinity range and were found at relatively low water temperatures in spring, whereas records associated with a high mean AIW (>5 g·ind^−1^) occupied a narrower salinity range and were linked to habitats with higher SBTs. In summer, records with low and high mean AIWs overlapped fully along salinity and temperature gradients (Table 3 and Table 4, Figure 6).

*Solenocera koelbeli* was distributed across depths of 18–110 m. It rarely occurred in extremely shallow waters less than 40 m, and most individuals concentrated at depths of 50–100 m. There were considerable inter-annual differences in spring: extremely high values were recorded at Wentai and Yushan under SSTs of 16–24 °C. In summer (August–September), CPUE remained low in most fishing grounds even when SST was above 28 °C, suggesting that this species does not favor high water temperatures in summer. In autumn–winter, fishing grounds with high CPUE had SSTs of 15–24 °C and SBTs of 15–22 °C. Catch records associated with a high mean AIW were observed at SBTs of 15–19 °C in winter, and records associated with a low mean AIW had a minimum recorded SBT of 15 °C. Most cohorts were found in high-salinity waters with SBS > 34. Catch records associated with a low mean AIW (<1 g·ind^−1^) occurred only sporadically in high-salinity waters in spring and summer. Catch records associated with a high mean AIW (>5 g·ind^−1^) spanned wider ranges of SBS and SBTs in spring and were recorded at water temperatures up to 28 °C in summer (Table 3 and Table 4, Figure 6).

## 4. Discussion

### 4.1. Life History Trait of Both Solenocera Shrimp Species

*Solenocera crassicornis* is a nearshore shrimp species. This species can be found in water areas influenced by Yangtze River diluted water. *Solenocera koelbeli* was observed to occur in deeper offshore waters with high salinity and reached its CPUE_w_ peak in autumn–winter and late spring, avoiding estuarine low-salinity waters. *S. koelbeli* could occupy waters deeper than 100 m where the SBT is consistently above 15 °C. *S. crassicornis* occur over a broad depth range (7–145 m) and is most frequently encountered in shallow waters < 40 m, with broad thermal tolerance down to ~7 °C. In spring, the main cohorts of *S. koelbeli* occupied habitats with significantly higher SBTs than those of *S. crassicornis*.

The spawning period of *S. crassicornis* was suggested to occur from June to October, with a peak in August to October [6]. In the Mindongbei fishing ground, the gonadal maturity stage composition of *S. crassicornis* was 38.8% at stage III in May; 48.7% at stage III and 16.2% at stage IV in August; 86.6% at stage II in November; and 80% at stage II in February [13]. The spawning period of *S. koelbeli* was from July to November, peaking in August–September [12]. The maximum body length of *S. koelbeli* was observed from June to September, while the minimum occurred from February to April [9]. In our study, the spawning period and peak timing of *S. koelbeli* were one month later than those of *S. crassicornis*.

In the East China Sea, recruitment of *S. crassicornis* individuals with body lengths of 30–40 mm after September increased month by month [6]. From September to the following January, the residual cohort gradually declined, while juvenile recruitment steadily increased [6]. The residual cohort attained its maximum body length in November [6]. In Mindongbei, mature parent individuals entered from offshore deep waters into coastal shallow waters for spawning, and their juveniles fed and grew in coastal shallow waters in August [13]. By November, the juveniles gradually moved toward offshore deep waters [13]. In the present study, we assumed that the parent cohorts could be found at Yushan and Zhoushan during the peak spawning in August. By January, most recruitment was concentrated north of 30° N, showing a potential northward distribution shift, with larger recruits being found in the Yangtze River Estuary for nursery.

Song et al. (2006) suggested that in the East China Sea, after spawning begins in July, juveniles of *S. koelbeli* with body lengths of 25–40 mm appeared in the fishing stock from August to February of the following year [9]. The juvenile cohort grew rapidly, reaching 80–115 mm in August–September, and the post-spawning broodstock became mature and died [9]. After October, the standing stock was replaced by the juvenile cohort [9]. In our study, spawning-ready broodstock of *S. koelbeli* were assumed to be found in the coastal water area of Mindong and Yushan in spring (May). The parent and juvenile recruitment cohorts remained in Yushan and Wentai during summer (August—the peak of the spawning period). The juvenile recruitment tended to move northward from Mindong to Yushan for the potential nursery. In autumn (November—the end of the spawning period) and winter, the recruitment remained in Yushan for potential nursery, with a small portion of the other recruits distributed sporadically across Jiangwai, Zhoushan, Zhouwai, Wentai, and Mindong. Historically, in the Central and Southern Zhejiang coastal waters during spring (March–April), the shrimp stock underwent nursery and reproductive migrations from deep waters toward inshore areas, moving from southeast to northwest [12]. From July to September, most *S. koelbeli* broodstock was concentrated around waters 60–70 m deep for spawning [12]. Starting in October, as cold air frequently shifted southward, coastal water masses gradually intensified, and the Taiwan Warm Current weakened, causing sea surface temperature to drop to 13–15 °C [12]. Subsequently, *S. koelbeli* gradually migrated back to deep waters for overwintering [12].

It is important to distinguish three related but distinct concepts in this study: statistical environmental associations, realized habitat use, and physiological tolerance. Our model outputs identify statistical associations between environmental variables and shrimp occurrence, which reflect realized habitat use observed from field surveys. These correlative patterns cannot be regarded as direct evidence of physiological preference or physiological tolerance. Definitive conclusions regarding physiological tolerance require controlled laboratory experiments, which are beyond the scope of this field-based modeling study.

### 4.2. Spatiotemporal Patterns and Management Implications of the Two Solenocera Shrimp Species

Both *Solenocera* shrimp exhibit strong seasonal and inter-annual fluctuations. *S. crassicornis* showed seasonal distributional shifts in depth distribution, individuals dispersed into shallower waters in summer, and the range of capture depths broadened markedly in winter. We observed abundant juveniles and high CPUE_w_ for *S. koelbeli* in autumn and winter, followed by summer, with the lowest biomass occurring in spring. Both species exhibited temporal offsets in seasonal occurrence and habitat depth, facilitating niche partitioning and reducing intense resource competition within the same sea area.

Spatially, the Zhoushan, Yushan, and Wentai fishing grounds serve as shared core habitats for the two *Solenocera* species. Nevertheless, the Dasha–Yangtze River Estuary fishing grounds in the Northern East China Sea mainly support *S. crassicornis*, whereas *S. koelbeli* is virtually absent from these waters. The spatial CPUE_w_ distribution pattern of *S. crassicornis* followed the gradient: Northern East China Sea (31–33° N) > Southern East China Sea (26–28° N) > Central East China Sea (28–31° N) [6]. *S. koelbeli* in the East China Sea was found at 26.25–27.75° N, 121.25–124.75° E (Mindong and Wentai) with the highest CPUE_w_ of 8091.8 g·h^−1^ in spring; 28.25–28.75° N, 122.75–123.25° E (Zhoushan), with 9299.1 g·h^−1^ and offshore of the Minjiang Estuary at 26–27° N, 120.75–121.25° E, with 131.34 g·h^−1^ in summer; this species was mainly distributed in Zhoushan, Zhouwai, Jiangwai, Yushan, Wentai, and Mindong at depths of 80–100 m, and was extremely rare in waters north of 31.5° N [14]. Song et al. (2006) suggested that *S. koelbeli* was absent from the Yangtze River Estuary and waters north of the Yangtze River Estuary (salinity < 33), as well as in the coastal waters of Zhejiang and Fujian [9].

Based on our survey observations, correlative HSI model outputs, and previously published literature, we put forward tentative research-based recommendations for fishery management, rather than definitive regulatory measures. For recruitment-stage shrimp stocks, the period from December to the following February represents a key growth window. Following the spawning peak (August–October), residual adult cohorts of *S. crassicornis* decline substantially, while juvenile recruitment rises [6]. From December to February, a large proportion of captured individuals are small-bodied shrimp with a body length < 50 mm, accounting for 43.8% of catches in January, 23.4% in December, and 26.2% in February [6]. Therefore, protection during December–February may benefit subsequent summer–autumn resource abundance.

*Solenocera koelbeli* constitutes an important target species for shrimp trawl fisheries in the South–Central East China Sea [15,16]. Historically, its spring fishing season in South–Central Zhejiang ran from April to June targeting feeding and recruitment stocks, whereas the summer–autumn fishing season (July–October) mainly targeted reproductive and feeding stocks, contributing 80% of the total catches [12]. Currently, the East China Sea summer fishing moratorium runs from 12:00 on May 1 to 12:00 on September 16 within Zhejiang waters (26.5–30° N) [17]. Most fishing operations are prohibited during this period, although licensed beam shrimp trawling is permitted starting at 12:00 on August 5 [16]. December–February is regarded as a critical growth period for juveniles [18]; over 70% of trawl catches in this window consist of small juveniles < 70 mm body lengths [12].

Our results suggest that the resource center of *S. crassicornis* has shifted toward the Yushan and Zhoushan fishing grounds, and the Yangtze River Estuary functions as a potentially important nursery ground in autumn and winter. For *S. koelbeli*, Mindong and Yushan are hypothesized to be potential spawning grounds, and Yushan may serve as a nursery ground for recruits. Accordingly, we tentatively recommend seasonal attention for broodstock from June to September in the Yushan, Zhoushan, and Mindong fishing grounds, alongside protection for recruitment stocks across December–February. It is also worthwhile to monitor how the Taiwan Warm Current shapes the spatial distributions of both shrimp species in the East China Sea. We emphasize that these specific time windows and geographical priority areas are partially hypothesis-driven, especially for the inferred spawning grounds; practical fishery implementation requires further empirical validation.

Collectively, niche partitioning between the two species may reduce the potential for interspecific competition. Model outputs show correlative trends of habitat contraction and southward boundary shifts; causal drivers cannot be disentangled based on current observational data. Future studies should focus on how long-term changes in bottom-water temperature and salinity alter habitat suitability of these *Solenocera* species under climate change, and separate the contributions of environmental forcing and fishery exploitation to population dynamics.

### 4.3. Study Limitations

Although this study uses fishery-independent bottom-trawl survey data, several important limitations should be acknowledged, which are categorized below into modeling-related, sampling-data, analytical-metric, ecological-inference, and trend-driver limitations.

First, the hybrid Tweedie GAM-BRT models exhibit robust temporal predictive performance but relatively poor spatial generalizability. Several critical environmental covariates, including prey abundance, seabed sediment type, oceanographic fronts, and fishing pressure, were not incorporated, thereby introducing prediction uncertainty. Cross-validation via partitioning the existing dataset cannot substitute for fully independent external validation, and suitable separate unused survey datasets for these shrimp species in our study area are currently unavailable. The two-dimensional thin-plate spline for longitude–latitude within the Tweedie GAM captures broad-scale spatial structure and spatial autocorrelation. Consequently, high AUC-ROC values may partly reflect sampling-driven spatial patterns rather than purely representing species–environmental niche signals. Under this penalized hybrid modeling framework, reliable quantitative variance partitioning between spatial coordinates and environmental predictors cannot be achieved; model outputs may partly reproduce observed spatial patterns instead of isolating the fundamental environmental niche, and formal variance-decomposition approaches are recommended for future work. Spatial cross-validation indicates limited spatial transferability, meaning HSI outputs are only suitable for tentative habitat-management inference within our surveyed domain and should not be extrapolated to unsampled marine areas outside our survey coverage. Temporal cross-validation, by contrast, demonstrates temporally stable species–environment associations. For species with sparse occurrence records, high tree complexity may capture idiosyncratic sampling noise alongside true ecological signals, and future work will compare a grid of tree complexity values (e.g., 3, 5, 10, 20) to explicitly test sensitivity of HSI outputs to this tuning parameter. BRT relative influence should not be interpreted as the fraction of deviance uniquely explained by each predictor.

Second, sampling coverage was inconsistent for nominal 30-arcminute grid cells across years and seasons. Pre-planned stations could be missing due to COVID-19 restrictions, motor-trawl prohibitions, bad weather, and navigation-safety constraints. Even under standardized survey protocols, trawl-based CPUE suffers from spatiotemporal variability in catchability and size-dependent gear selectivity. *Solenocera koelbeli* has far fewer occurrence records compared with *S. crassicornis,* and limited positive observations may exaggerate apparent environmental specialization in model outputs. Field and historical literature support its preference for deep, high-salinity offshore habitats, yet sample-size-driven artifacts cannot be fully excluded; additional surveys with more positive catches are required to clarify its niche characteristics. Body-length measurements were only collected from partial survey cruises due to offshore logistical constraints. Inferences on the population-size structure rely on these partial subsets, and we lack independent benchmarks confirming that sampled size spectra fully represent wild populations. Station-level average individual weight (AIW) is an aggregated trawl-station metric rather than individual biological measurement, so AIW thresholds cannot reliably distinguish biological juvenile and adult shrimp stages.

Third, AUC-ROC was our primary metric for evaluating discriminative performance. Nevertheless, AUC-ROC can be misleading under uneven species prevalence, strong spatial autocorrelation and biased background sampling. The relatively low spatial cross-validation AUC-ROC of *S. crassicornis* indicates limited capacity to predict its distribution in new, unsampled spatial locations. Supplementary classification diagnostics (sensitivity, specificity, balanced accuracy, Brier score, model-calibration metrics) and bootstrap-derived confidence intervals for AUC-ROCs were not computed in our present workflow, and these would offer deeper insights in future analyses. Our cross-validation strategy ensures that BRT weights are re-estimated within each CV fold, preventing information from test observations from leaking into the weight calculation.

Fourth, observed seasonal distributional shifts cannot unambiguously demonstrate active shrimp migration. Patterns may alternatively arise from spatially heterogeneous recruitment, differential regional mortality, cohort turnover, or inter-cruise sampling variation. The habitat–environment relationships reported here are correlative, not causal. Several key phenological windows are mainly derived from the prior literature rather than via statistical segmentation of our dataset. Comparisons of habitat suitability across fishing grounds are based on HSI point estimates with no adjustment for prediction uncertainty. We observe clear compartmentalization of environmental, spatial, and seasonal niches between the two *Solenocera* species, alongside partial spatial overlap within core fishing grounds. This niche-partitioning pattern generates the plausible hypothesis that such differences may reduce potential interspecific competition, yet this hypothesis remains untested in our study. Rigorous tests for competition require quantitative niche-overlap indices, together with morphometric comparisons between sympatric and allopatric populations, to evaluate character displacement, which cannot be achieved using our current dataset. Uncertainty was quantified separately for each model component: single-factor suitability indices were accompanied by approximate 95% confidence intervals derived from the GAM linear-predictor standard errors, while BRT prediction uncertainty was assessed through 600 bootstrap replicates, summarized as bootstrap mean, standard deviation and empirical 95% percentile intervals. However, these two sources of uncertainty were not formally propagated into a single uncertainty estimate of the composite HSI, and covariance among SI components and BRT weights was not considered. The composite HSI should therefore be treated as a point estimate rather than an interval estimate of habitat suitability.

Fifth, the anomalous high-catch event recorded in November 2021 cannot be mechanistically disentangled given the constraints of our time-series dataset. Annual areal fractions of highly suitable habitat are HSI-derived point estimates; bootstrapped confidence intervals were not generated, so we cannot statistically confirm whether the apparent downward trend in suitable habitat for *S. crassicornis* is significant. Formal statistical trend testing conditioned on year, season and sampling effort is not feasible given our unbalanced field-survey dataset. Because fishing pressure, sediment characteristics, prey availability and oceanographic fronts are absent from our models, habitat contraction and southward shifts in northern boundaries cannot be uniquely attributed to nearshore environmental change. Competing plausible drivers include shifts in fishery exploitation, benthic habitat alteration, prey-resource variation and inter-annual sampling heterogeneity. These competing hypotheses cannot be disentangled using the existing data and require multi-source datasets for future investigations.

## 5. Conclusions

Our conclusions can be summarized as follows:

(1) The Tweedie GAM-BRT framework exhibits reliable temporal predictive performance for shrimp habitat suitability, whereas spatial generalization capacity is comparatively limited.
(2) Modeled and field-observed patterns show that the two *Solenocera* shrimp species exhibit distinct environmental niches.
(3) Field and model outputs suggest niche partitioning by season and body size, which may mitigate interspecific competition.
(4) Observed patterns of contracting highly suitable habitats and southward shifts in northern habitat boundaries represent correlative spatiotemporal trends in our dataset; causal drivers behind these trends cannot be disentangled using the present survey data.

## Figures and Tables

**Table 1:** Information of field surveys (cruise name, investigation period, and season), the number of sampling stations, and the number of positive stations for *Solenocera crassicornis* and *Solenocera koelbeli*.

No	Cruise Name	Investigation Period	Season	Number of Sampling Stations	*S. crassicornis*	*S. koelbeli*
					Number of Positive Stations	
1	2018–11	2–11 November 2018	autumn	127	38	22
2	2019–01	4–27 January 2019	winter	111	33	26
3	2019–05	22 April–10 May 2019	spring	141	0	7
4	2019–08	13 August–27 September 2019	summer	140	19	10
5	2020–05	7 May–17 June 2020	spring	143	5	7
6	2020–08	13 August–27 September 2020	summer	140	35	4
7	2021–05	22 April–19 June 2021	spring	87	5	4
8	2021–11	2 November 2021–4 January 2022	autumn	105	35	18
9	2022–06	20–28 June 2022	spring	42	4	2
10	2022–09	25 August–28 September 2022	summer	90	11	3
11	2022–12	6–28 December 2022	winter	53	26	3
12	2023–05	11 April–6 June 2023	spring	146	9	2
13	2023–08	16 August–20 September 2023	summer	155	12	3
14	2023–11	4 November–3 December 2023	autumn	59	7	7
15	2023–12	24 December 2023–6 January 2024	winter	54	11	17
16	2024–04	11 April–21 May 2024	spring	157	11	15
17	2024–09	25 August–27 September 2024	summer	75	5	0
18	2024–12	11 November 2024–23 January 2025	winter	115	32	16
19	2025–04	19 April–26 May 2025	spring	157	14	9
20	2025–08	5 August–12 September 2025	summer	155	8	10
21	2025–11	22 November 2025–15 January 2026	winter	120	35	10

Note: Cruise name represents the survey voyage code (survey year–month); survey period indicates the actual sampling date; total station number is the total number of sampling stations in each voyage; and catch station number represents the number of stations where the target species is captured.

**Table 2:** Monthly total values and value ranges of CPUE_w_ (g·h^−1^), CPUE_n_ (ind·h^−1^), and AIW (g·ind^−1^) of *Solenocera crassicornis* and *S. koelbeli* from November 2018 to November 2025.

Month	*Solenocera crassicornis*					*Solenocera koelbeli*				
	Total Value		Value Range			Total Value		Value Range		
	CPUE_w_	CPUE_n_	CPUE_w_	CPUE_n_	AIW	CPUE_w_	CPUE_n_	CPUE_w_	CPUE_n_	AIW
Nov, 2018	4398.5	2023.6	2.1–904.1	1.3–305.5	0.5–5.7	13,925.1	3964.9	2.9–7111.2	2–2064	0.5–13.7
Jan, 2019	2123.6	1244	2.4–223.7	1.2–154.3	0.5–7.1	18,698.3	2815.4	4.7–4070.4	2–624	0.6–48.8
May, 2019	no catch	no catch	no catch	no catch	no catch	1198.4	148.8	3.5–500.5	1–60.9	3.5–9.5
Aug, 2019	10,108.2	2751.2	31.9–2702.4	8–825	2–10.8	5699.3	975	1.7–1716	1–286	1.7–9.8
May, 2020	391.2	96	3.7–230.4	2.7–48	1.4–4.8	4159.2	699.7	33.2–1453.6	3.7–416	3.5–10.7
Aug, 2020	21,743.2	15,457.5	0.4–4163.2	1.2–4896	0.3–10.8	1825	381.7	12–1209.6	1.5–314.2	3.9–9.2
May, 2021	355	97.3	6.4–169.8	8.1–32	0.4–6.3	152.5	32.8	15.6–99.9	3–16	1–9.3
Nov, 2021	23,449.8	9425.9	1.8–12,360	3–3600	0.5–24.4	78,652.3	20,455.4	3.5–46,912	4.1–10,560	0.4–5.8
Jun, 2022	1075.1	683.5	0.4–565.7	4–504	0.1–3.5	2642	496	382.8–2259.2	48–448	5–8
Sep, 2022	9937.2	8672	22.4–2985.6	21.8–4352	0.6–7.5	893.1	113	8.3–520	1–80	6.5–11.4
Dec, 2022	2072.6	1116.6	2.2–295.2	3.7–134.4	0.3–3.7	255.1	637.3	6.7–211.2	2.4–630.9	0.3–9.3
May, 2023	564.4	296.9	19.2–171.2	3.7–67.2	0.9–7.8	248.8	216.4	64.9–183.9	54.1–162.3	0.4–3.4
Aug, 2023	4582.3	2669.6	0.3–1531.2	1–960	0.1–9.2	2806.6	424.6	378–1366.2	112–184.6	3.4–8.3
Nov, 2023	1385.8	758.4	19.2–745.6	8–448	0.5–9	4749.9	1573.8	12.5–3361.9	9.6–1056	1.3–4.4
Dec, 2023	2848.5	1133.2	4–880	4–352	0.4–5.5	13,216.1	3024.5	27.5–3252	4.4–680	0.4–15.7
May, 2024	1924.3	995.7	1–1008	1–560	0.8–3.1	10,533.4	1710.9	8.7–6400.1	1–921.9	1.2–10
Sep, 2024	1414.3	796	14.8–522	2–260	1.3–7.4	no catch	no catch	no catch	no catch	no catch
Dec, 2024	7717.8	2043.3	0.7–2592	1–608	0.6–7.1	7622.3	1550.5	4.5–2116	2–408	1.8–7
May, 2025	1238.6	512.3	0.9–435.2	1–208	0.3–9.4	12,531.2	1681	10.4–7318.4	1–896	2.1–10.4
Aug, 2025	3223.1	1412.1	2–1862.4	4–1024	0.5–6.9	6251.1	1666	3–2678.4	5–864	0.6–11.9
Nov, 2025	2150.8	1134.8	0.3–335.2	1–192	0.3–6.3	3253.5	1018.7	9.6–1564.3	4–629.5	1.4–10

**Table 3:** Value ranges of environmental variables (SST, SSS, SBT, SBS, and depth) at stations where *Solenocera crassicornis* and *S. koelbeli* were captured from November 2018 to November 2025.

Month	*Solenocera crassicornis*					*Solenocera koelbeli*				
	SST	SSS	SBT	SBS	Depth	SST	SSS	SBT	SBS	Depth
Nov, 2018	18–23.6	31–34	12.3–22.8	30–35	14–76	21.2–23.9	32–35	17.5–22.6	34–35	51–104
Jan, 2019	10.7–21.9	32–35	10.8–19.8	31–35	29–145	14.9–19.6	33–35	15.2–19.4	33–35	46–100
May, 2019	no catch	no catch	no catch	no catch	no catch	17.2–24.8	30–35	15.4–22.8	33–35	54–98
Aug, 2019	25.1–29.5	16–34	11.2–27.7	30–35	16–75	26–28.9	31–34	17.3–28	33–35	69–97
May, 2019	21.8–25	30–33	13.5–20.9	32–34	35–58	19.4–25.3	31–34	17.2–23.7	34–35	33–68
Aug, 2020	24.1–29.2	13–34	17.8–26.8	14–35	7–94	28.4–29.5	30–34	20.7–26	34–35	54–64
May, 2021	18.4–24.8	30–34	17.2–23.1	33–35	27–85	20.6–23.6	29–34	18.1–23.1	30–35	24–81
Nov, 2021	9.8–25.7	30–34	9.9–24.4	30–34	21–71	18.6–25.7	30–34	18.6–24.7	32–35	46–97
Jun, 2022	23.3–25.1	24–30	17.6–21.5	31–35	27–47	23.5–23.9	29–31	18.2–22.6	31–34	18–54
Sep, 2022	24.6–28	29–34	21.7–26	30–34	24–57	25.4–26.6	34–35	19.5–22.1	34–35	62–91
Dec, 2022	13.4–18.5	31–34	13.5–19.5	31–34	25–84	15–18.3	33–34	16–18.5	33–34	36–56
May, 2023	14.5–20.3	31–34	13.4–18.6	32–34	25–71	19–20.3	34–35	18.2	34–35	70–83
Aug, 2023	27.4–29.9	28–34	18.5–24.5	32–35	37–104	29–29.5	34–35	21.8–24.7	34–35	51–66
Nov, 2023	20.2–23.8	28–34	17.9–22.1	34–35	36–90	18.3–23.7	30–36	17.9–22.1	34–35	54–93
Dec, 2023	11.8–20.1	25–35	13–18.1	30–35	20–113	14.7–19.8	31–35	17.4–19.6	33–34	40–89
May, 2024	14.1–24.1	28–34	12.3–19.6	32–35	47–85	13.4–24.1	28–34	13–20.9	33–35	44–85
Sep, 2024	29.1–30.3	30–33	21.2–25.5	33–34	35–70	no catch	no catch	no catch	no catch	no catch
Dec, 2024	10.3–23.4	30–34	10.5–23.4	30–34	24–99	15.8–24.6	30–35	16–24	30–35	24–110
May, 2025	14.5–22.5	31–34	10.6–20.8	32–34	22–58	16.3–24.8	32–34	15.7–22.2	34–35	51–100
Aug, 2025	27.2–30.6	33–34	18.6–26.3	33–35	23–113	29–30.8	33–34	18.7–22.8	34–35	50–101
Nov, 2025	7.4–22.1	29–35	7.5–22.1	29–34	12–91	16.9–23.1	21–35	16.9–22.5	34–35	41–100

Note: SST, sea surface temperature; SSS, sea surface salinity; SBT, sea bottom temperature; SBS, sea bottom salinity—“no catch” indicates that the species was not collected in this survey cruise.

**Table 4:** Variations in SBS and SBTs of *Solenocera crassicornis* and *S. koelbeli* across seasons under different CPUE_w_ and AIWs.

Season	Type	*Solenocera crassicornis*		*Solenocera koelbeli*	
		SBS	SBT	SBS	SBT
spring	500–1000 g·h^−1^	32–34	12–22	34–35	20–23
summer	500–1000 g·h^−1^	29–35	19–26	34–35	21–22
autumn	>1000 g·h^−1^	33–35	19–23	33–35	18–23
winter	500–1000 g·h^−1^	33	17	33–35	16–22
spring	<1 g·ind^−1^	30–35	13–22	35	18
summer	<1 g·ind^−1^	29–35	18–26	34–35	20–21
autumn	<1 g·ind^−1^	32–35	17–23	32–35	17–22
winter	<1 g·ind^−1^	31–35	8–22	33–34	15–18
spring	>5 g·ind^−1^	32–34	17–23	30–35	13–24
summer	>5 g·ind^−1^	29–35	18–26	31–35	18–28
autumn	>5 g·ind^−1^	31–35	16–22	34–35	17–22
winter	>5 g·ind^−1^	31–34	12–24	32–35	15–21

## Data Availability

The original contributions presented in this study are included in the article. Further inquiries can be directed to the corresponding authors.

## Supplementary Materials

The following supporting information can be downloaded at: https://www.mdpi.com/article/10.3390/biology15211950/s1, Figure S1: Residual diagnostic plots for the generalized additive models of environmental variables, such as sea surface temperature, sea surface salinity, sea bottom temperature, sea bottom salinity, and depth, for shrimp species *Solenocera crassicornis* and *Solenocera koelbeli*. Each group contains four subgraphs: deviance residuals against linear predictors, Q-Q normal quantile plots, response versus fitted values, and histograms of residuals; Figure S2: Habitat suitability index (HSI) density distributions for two shrimp species: *Solenocera crassicornis* and *Solenocera koelbeli*. Density curves are separated by observation status (presence vs. absence) to illustrate differences in HSI value frequency between sampling sites with and without target shrimp occurrence; Figure S3: Length–frequency distributions of *Solenocera crassicornis* and *Solenocera koelbeli* from different sampling sites, months, and years. Notes: Red dashed lines indicate mean body length; N is sample size; ♀:♂ represents female to male sex ratio; and roman numerals (I V) denote gonadal development stages; Figure S4: Spatiotemporal variations in habitat suitability index (HSI) for *Solenocera crassicornis* (left panels) and *S. koelbeli* (right panels) across the study area from November 2018 to November 2025. Color gradients represent HSI values ranging from zero to one; higher values indicate more suitable habitats for the two shrimp species. No catch of *S. crassicornis* was recorded in May 2019, and no catch of *S. koelbeli* in September 2024. Thus, data from these two months are excluded from the results; Table S1: Boosted regression tree (BRT) model parameters and predictive performance for Solenocera crassicornis and *Solenocera koelbeli*; Table S2: Variable relative influence and model diagnostic statistics of boosted regression tree (BRT) models for *Solenocera crassicornis* and *Solenocera koelbeli*; Table S3: Temporal held-out validation performance per held-out year for *Solenocera crassicornis* and *Solenocera koelbeli*; Table S4: Mean and total values of catch per unit effort in weight (CPUEw) and weight percentage (CPUEw%), catch per unit effort in number (CPUEn) and number percentage (CPUEn%), and average individual weight (AIW), together with environmental conditions [sea surface temperature (SST), sea surface salinity (SSS), sea bottom temperature (SBT), sea bottom salinity (SBS), and depth], for *Solenocera crassicornis* and *Solenocera koelbeli* across survey seasons and fishing grounds during 2018–2025.
