# Supplementary material for: Habitat Suitability and Environmental Niche Differentiation of Two Commercially Important Solenocera Shrimp Species in the Southern Yellow Sea and East China Sea (2018–2026)
Source: Biology (Basel). 2026 Oct 29;15(21):1950. doi: 10.3390/biology15211950 (PMC13761788; doi:10.3390/biology15211950)
Supplement: Supplementary file 1 [file biology-15-01950-s001.zip › biology-4576207-supplementary.pdf]

Supplementary file S1

Table S1. Boosted regression tree (BRT) model parameters and predictive performance for *Solenocera crassicornis* and *Solenocera koelbeli*.

Parameter	*Solenocera crassicornis*	*Solenocera koelbeli*
Hold-out number of test observations	699	699
Hold-out area under the receiver-operating-characteristic curve for presence-absence data	0.7776	0.8551
Hold-out normalized root-mean-square error	0.6778	0.8788
Temporal-CV mean AUC-ROC	0.7565	0.8789
Temporal-CV SD-AUC-ROC	0.0958	0.0324
Temporal-CV mean NRMSE	0.7044	0.8416
Temporal-CV SD-NRMSE	0.0503	0.0247
Spatial-CV mean AUC-ROC	0.5921	0.6901
Spatial-CV SD AUC-ROC	0.1652	0.2054
Spatial-CV mean NRMSE	0.6434	0.8412
Spatial-CV SD-NRMSE	0.1263	0.165
RMSE	91.3612	81.5409
AUC-ROC	0.7953	0.8836
Cross-validation folds	20	20
Tree complexity	5	5
Learning rate	0.01	0.01
Bag fraction	0.6	0.6
Optimal number of trees	200	200

Note: Temporal-CV mean AUC-ROC, temporal cross-validation mean area under the receiver-operating-characteristic curve for presence-absence data; Temporal-CV SD-AUC-ROC, temporal cross-validation standard deviation of area under the receiver-operating-characteristic curve for presence-absence data; Temporal-CV mean NRMSE, temporal cross-validation mean normalized root-mean-square error; Temporal-CV SD-NRMSE, temporal cross-validation standard deviation of normalized root-mean-square error; spatial-CV mean AUC-ROC, spatial cross-validation mean area under the receiver-operating-characteristic curve for presence-absence data; spatial-CV SD AUC-ROC, spatial cross-validation standard deviation of area under the receiver-operating-characteristic curve for presence-absence data; spatial-CV mean NRMSE, spatial cross-validation mean normalized root-mean-square error; spatial-CV SD-NRMSE, spatial cross-validation standard deviation of normalized root-mean-square error; RMSE, root-mean-square error; AUC-ROC, area under the receiver-operating-characteristic curve for presence-absence data.

Table S2. Variable relative influence and model diagnostic statistics of boosted regression tree (BRT) models for *Solenocera crassicornis* and *Solenocera koelbeli*.

Factor	Relative influence	Deviance explained	AUC-ROC	Tweedie scale parameter	Residual degrees of freedom	Concurvity		
						Worst-case	Observed-case	Estimated-case
Solenocera crassicornis								
SST	21.9164	0.3953	0.7993	34.1309	1529.0856	0.1266	0.116	0.1297
SSS	12.6835	0.3963	0.7934	34.0688	1528.5542	0.1047	0.1911	0.139
SBT	19.8574	0.3912	0.7873	34.2758	1529.7722	0.2263	0.1802	0.1353
SBS	18.5959	0.3896	0.7895	34.3586	1529.4616	0.1591	0.1774	0.1258
Depth	14.0509	0.3888	0.789	34.379	1529.6021	0.2816	0.1727	0.1303
Solenocera koelbeli								
SST	13.9216	0.6404	0.9019	35.6743	1583.0109	0.1212	0.1756	0.1104
SSS	9.3071	0.6469	0.9033	35.2632	1582.0119	0.1826	0.1819	0.1263
SBT	10.8552	0.644	0.8989	35.3597	1583.2041	0.2345	0.1792	0.1255
SBS	23.8164	0.6425	0.9015	35.4652	1582.1467	0.2524	0.1612	0.164
Depth	27.2163	0.6265	0.8907	36.425	1586.1211	0.1813	0.1395	0.1253

Note: area under the receiver-operating-characteristic curve for presence-absence data.

Table S3. Temporal hold-out validation performance per held-out year for *Solenocera crassicornis* and *Solenocera koelbeli*.

	Solenocera crassicornis		*Solenocera koelbeli*	
Held-out-year	Number of test observations	AUC-ROC	Number of test observations	AUC-ROC
2018	88	0.9107	88	0.8822
2019	179	0.7641	280	0.843
2020	198	0.7956	197	0.9263
2021	134	0.873	133	0.8548
2022	128	0.7554	128	0.8504
2023	288	0.7833	288	0.8703
2024	240	0.747	188	0.8768
2025	301	0.7232	302	0.9273

Note: area under the receiver-operating-characteristic curve for presence-absence data.

Table S4. Mean and total values of catch per unit effort in weight ($CPUE_w$), weight percentage ($CPUE_w$%), catch per unit effort in number ($CPUE_n$), number percentage ($CPUE_n$%), and average individual weight (AIW), together with environmental conditions [sea surface temperature (SST), sea surface salinity (SSS), sea bottom temperature (SBT), sea bottom salinity (SBS), and depth], for *Solenocera crassicornis* and *Solenocera koelbeli* across survey seasons and fishing grounds during 2018–2025.

	Mean value					Total value				Environmental Factors				
Fishing ground	$CPUE_w$		$CPUE_n$		AIW	$CPUE_w$		$CPUE_n$		SST	SSS	SBT	SBS	Depth
	Solenocera crassicornis													
	November 2018 (Autumn)													
Lvsi	75.1	9.2%	22.6	6.1%	3.5	150.3	3.4%	45.1	2.2%	18–18.4	31–32	18.1–18.4	32	14–28

Dasha	82.5	10.1%	38.2	10.3%	2.2	660.4	15%	305.6	15.1%	18.2–21.2	31–33	12.3–20	30–33	28–47
Yangtze River Estuary	58.6	7.2%	25.4	6.9%	2.1	410.5	9.3%	177.9	8.8%	19.9–21.7	32–34	19.9–21.7	32–34	35–44
Jiangwai	78.4	9.6%	45.5	12.3%	1.7	470.7	10.7%	273.2	13.5%	19.9–22.6	33–34	19.9–22.7	32–34	46–69
Zhoushan	230	28.2%	89.6	24.3%	2.9	1379.9	31.4%	537.5	26.6%	21.5–22.3	33–34	21.6–22.3	33–34	41–60
Zhouwai	122.6	15.1%	55.4	15%	2.1	490.5	11.2%	221.8	11%	22.2–22.7	33–34	22.1–22.7	33–34	57–69
Yushan-Wentai	167.2	20.5%	92.5	25.1%	2	836.2	19.0%	462.4	22.9%	22.1–23.6	32–34	20.3–22.8	34–35	47–76
January 2019 (Winter)														
Dasha	71.7	19.4%	47.8	21.7%	1.6	502.2	23.6%	334.4	26.9%	10.7–13.6	31–33	10.8–14	31–34	29–54
Yangtze River Estuary	71.7	19.4%	41	18.6%	2.9	429.9	20.2%	245.8	19.8%	11.6–13.5	32–34	11.9–13.7	32–34	35–42
Jiangwai	89	24.1%	54.4	24.7%	3.1	355.9	16.8%	217.7	17.5%	13.2–18.1	33–34	13.5–18.3	33–35	48–90
Zhoushan	47.3	12.8%	21.3	9.7%	2	331.4	15.6%	149.1	12%	14.3–16.6	33–35	14.4–16.7	33–35	41–66
Zhouwai	78.4	21.2%	42.9	19.4%	1.8	470.6	22.2%	257.1	20.7%	14.9–17.8	33–34	15–18.1	33–34	58–85
Mindong	11.2	3%	13.3	6%	0.8	33.6	1.6%	40	3.2%	17.6–21.9	34–35	18.3–19.8	34–35	50–145
August 2019 (Summer)														
Dasha	95.8	3.1%	32.5	3.9%	4.2	287.4	2.8%	97.5	3.5%	26.1–26.9	30–32	11.2–25.8	30–33	16–65
Yangtze River Estuary	305.6	9.9%	130.5	15.7%	4.5	916.8	9.1%	391.4	14.2%	27.2–28.4	16–32	19.9–23.8	32–35	18–44
Zhoushan	846.8	27.4%	251.1	30.1%	5.5	3387.1	33.5%	1004.3	36.5%	25.2–29.5	28–32	19.1–24.7	32–35	25–60
Yushan	1077.6	34.9%	251.6	30.2%	5.7	3232.8	32%	754.9	27.4%	26.8–28.3	29–33	20.8–27.6	31–35	31–55
Wentai	500.5	16.2%	92	11%	5.2	1501.6	14.9%	276	10%	25.1–28.3	30–34	25.6–27.7	32–34	43–59
Mindong	260.9	8.4%	75.7	9.1%	3.6	782.6	7.7%	227.2	8.3%	25.2–26.3	30–34	26.2–26.3	32–34	54–75
May 2020 (Spring)														
Dasha	230.4	58.9%	48	50%	4.8	230.4	50.4%	48	42.1%	21.8	30	13.5	32	48.3
Yangtze River Estuary	3.7	1%	2.7	2.8%	1.4	69.6	15.2%	20.8	18.2%	22.7–24.8	31–32	17.6–20.7	32–34	35–56
Zhoushan	139.2	35.6%	41.6	43.4%	6.8	139.2	30.5%	41.6	36.5%	25	32.9	20.8	35	57.7

Wentai	17.8	4.6%	3.7	3.8%	4.8	17.8	3.9%	3.7	3.2%	23	31.7	20.9	34	48.1
August 2020 (Summer)														
Dasha	297.2	8.2%	346.8	13.3%	1	3269.7	15.0%	3814.7	24.7%	24.1–27.1	27–31	23.4–26.8	29–34	14–55
Yangtze River Estuary	495.2	13.6%	285.7	11%	1.6	4456.9	20.5%	2570.9	16.6%	24.3–25	13–34	21–25.1	14–34	7–66
Zhoushan	940.8	25.8%	528.5	20.3%	1.8	5645	26%	3170.7	20.5%	24.1–25.8	26–33	21.6–26.5	30–34	24–44
Yushan	727.0	19.9%	141.8	5.5%	5.1	3634.8	16.7%	709.1	4.6%	28.3–29.2	33–34	17.8–23.4	34–35	31–94
Wentai	1184.2	32.5%	1298	49.9%	3.5	4736.8	21.8%	5192	33.6%	27.8–28.9	34–35	24.8–25.9	34–35	21–49
May 2021 (Spring)														
Zhoushan	70.7	49.8%	23.1	57.5%	3.5	141.4	39.8%	46.1	47.4%	18.4–19.8	30–31	17.2–18.3	33–34	27–51
Wentai	71.2	50.2%	17	42.5%	3.8	213.6	60.2%	51.1	52.6%	21.9–24.8	32–34	18–23.1	34–35	31–85
November 2021 (Autumn)														
Dasha	77.6	2.0%	31.7	2.1%	2.3	776.4	3.3%	317.1	3.4%	9.8–25.7	30–32	9.9–18.6	30–32	21–52
Yangtze River Estuary	79.5	2.0%	53.0	3.6%	2.7	954.2	4.1%	636.1	6.7%	16.1–18.4	31–33	16.2–18.4	31–33	29–68
Zhoushan	972.8	24.8%	499.4	33.8%	2.8	7782.4	33.2%	3995.3	42.4%	18.6–23.7	32–34	18.6–22.6	33–34	49–66
Yushan	2787.4	71.2%	895.5	60.5%	7.6	13936.8	59.4%	4477.3	47.5%	20.5–24.3	31–34	20–24.4	34–35	36–71
June 2022 (Spring)														
Yangtze River Estuary	7.2	0.7%	4.0	0.6%	1.8	14.4	1.3%	8.1	1.2%	23.3–24.8	24–30	17.6–18.4	31–32	33–37
Zhoushan	495.0	46.4%	504.0	74.2%	1.0	495.0	46.0%	504.0	73.7%	24.5	30	21.5	34	46.8
Yushan	565.7	53.0%	171.4	25.2%	3.3	565.7	52.6%	171.4	25.1%	25.1	30	19.5	34	27.2
September 2022 (Summer)														
Dasha	80.3	3.8%	97.3	5.1%	0.9	160.6	1.6%	194.6	2.2%	24.6–27.1	30–31	21.7–26	30–32	24–32
Yangtze River Estuary	518.9	24.3%	546.7	28.7%	0.9	2075.4	20.9%	2186.8	25.2%	25–28	29–34	21.7–24.1	33–34	36–54
Zhoushan	1540.2	72.0%	1258.1	66.1%	2.8	7701.2	77.5%	6290.5	72.5%	25.4–27	32–34	21.8–25.4	33–34	36–57
December 2022 (Winter)														

Dasha	72.0	29.9%	42.1	32.1%	1.7	936.1	45.2%	547.4	49.0%	13.4–18.4	31–34	13.5–18.7	31–34	25–84
Yangtze River etuary	98.0	40.7%	41.0	31.2%	2.2	783.7	37.8%	327.7	29.3%	16.4–18.5	32–34	17.2–19.5	32–34	27–51
Jiangwai	70.6	29.3%	48.3	36.8%	1.7	352.9	17.0%	241.5	21.6%	14–18.2	32–34	14.7–18.3	33–34	46–66
May 2023 (Spring)														
Zhoushan	35.8	18.4%	17.6	15.9%	2.1	143.4	25.4%	70.6	23.8%	14.5–18.9	33–34	13.4–14.5	34–35	45–71
Yushan	55.4	28.5%	53.7	48.4%	1	110.8	19.6%	107.4	36.2%	15.4–20.3	32–34	17.6–18.2	34–35	49–70
Wentai	103.4	53.1%	39.6	35.7%	4.3	310.2	55.0%	118.9	40%	16.7–18.2	31–32	15.2–18.6	32–34	25–49
August 2023 (Summer)														
Zhoushan	262.9	17.8%	114.1	14.9%	4.0	788.7	17.2%	342.2	12.8%	27.4–29.1	29–34	19.8–24.5	34–35	46–61
Zhouwai	378.7	25.7%	83.5	10.9%	5.3	1136.1	24.8%	250.5	9.4%	29–29.9	30–34	18.5–20.6	32–35	39–103
Yushan	496.3	33.7%	471.0	61.6%	0.7	1985.2	43.3%	1884.0	70.6%	28.1–29.5	28–34	19.9–21.8	34–35	41–76
Wentai	336.2	22.8%	96.5	12.6%	1.9	672.3	14.7%	193.0	7.2%	28.6–29.7	31–34	18.7–24.3	34–35	37–104
November 2023 (Autumn)														
Zhoushan	361.3	82.7%	162.7	70.6%	5.6	1083.8	78.2%	488.0	64.3%	23.6–23.8	28–30	19.2–20.6	34–35	36–54
Yushan	75.5	17.3%	67.6	29.4%	1.1	302.0	21.8%	270.4	35.7%	20.2–23.5	30–34	17.9–22.1	34–35	58–90
December 2023 (Winter)														
Zhoushan	502.4	55.4%	92.0	30.8%	5.4	1004.8	35.3%	184.0	16.2%	11.8–13.2	30–31	13.8–14.9	31–32	27–30
Yushan	227.2	25.1%	122.1	40.9%	2.3	1135.9	39.9%	610.4	53.9%	14–17.1	25–33	14.3–17.8	31–34	26–60
Wentai	177.0	19.5%	84.7	28.4%	1.9	707.9	24.9%	338.9	29.9%	12.8–20.1	30–35	13–18.1	30–35	20–113
April 2024 (Spring)														
Dasha	632.5	75.6%	335.5	77.7%	2.1	1265	65.7%	671	67.4%	14.1–16	32–34	12.5–12.6	32–34	47–74
Jiangwai	108.4	12.9%	56	13%	1.5	433.6	22.5%	224	22.5%	17.6–19.5	32–33	12.3–15.1	32–34	53–74
Zhoushan	62.9	7.5%	20.3	4.7%	2.1	125.7	6.5%	40.7	4.1%	14.3–15.7	29–34	14.9–17.3	34–35	54–55
Yushan	33.3	4.0%	20	4.6%	1.6	100	5.2%	60	6%	14.3–24.1	28–34	16.4–19.6	33–35	48–85

September 2024 (Summer)														
Jiangwai	282.9	100%	159.2	100%	3.7	1414.3	100%	796	100%	29.1–30.3	30–33	21.2–25.5	33–34	35–70
December 2024 (Winter)														
Dasha	49.2	3.1%	19.3	4.6%	1.9	196.7	2.5%	77.3	3.8%	10.3–13.6	31–34	10.5–13.9	31–34	36–61
Yangtze River Estuary	52.2	3.3%	13.7	3.3%	3.5	365.3	4.7%	95.7	4.7%	12–19.8	32–34	12.1–20	32–34	30–51
Jiangwai	71.2	4.5%	40.2	9.7%	1.8	355.9	4.6%	200.9	9.8%	11.7–15.6	32–34	12.2–15.5	32–34	43–77
Zhoushan	64.7	4.1%	18.2	4.4%	3.5	453.2	5.9%	127.4	6.2%	19.9–23.4	33–34	20.3–23.4	33–34	37–66
Yushan	327.9	20.9%	82.0	19.7%	3.8	1311.6	17.0%	328	16.1%	15.8–21.5	31–34	16.2–18.9	31–34	24–99
Wentai	1007.0	64.1%	242.8	58.3%	4	5035.1	65.2%	1214	59.4%	14.6–17.9	30–33	15–19.3	30–34	25–44
April 2025 (Spring)														
Dasha	3.3	0.9%	1.0	0.6%	3.3	3.3	0.3%	1.0	0.2%	14.5	33	10.6	34	60
Yangtze River Estuary	55.7	15.8%	23.9	15.2%	1.9	222.8	18.0%	95.7	18.7%	16.7–19.9	31–33	13–18.5	32–34	28–48
Zhoushan	19.5	5.5%	4.7	3.0%	2.6	58.4	4.7%	14.1	2.7%	15.6–16.2	31–32	15–15.5	32–33	24–38
Yushan	73.0	20.7%	54.8	34.8%	4.8	146.1	11.8%	109.7	21.4%	19.5–19.8	33–34	18.8–19.3	34–35	43–44
Wentai	202.0	57.1%	73.0	46.4%	4.6	808.0	65.2%	291.9	57.0%	18.8–22.5	32–34	17.5–20.8	33–34	22–51
August 2025 (Summer)														
Zhoushan	200.1	29.8%	78.7	27.3%	3.9	400.2	12.4%	157.4	11.1%	27.2–28.8	33–34	20.2–23	34–35	35–45
Wentai	470.5	70.2%	209.1	72.7%	3.3	2822.9	87.6%	1254.8	88.9%	27.9–30.6	33–34	18.6–26.3	33–35	23–113
November 2025 (Autumn)														
Dasha	29.1	6.1%	13.4	5.0%	1.9	349.4	16.2%	160.5	14.1%	7.4–19.2	31–34	7.5–19.1	31–34	12–91
Yangtze River estuary	5.8	1.2%	5	1.9%	1.6	29.0	1.4%	25.1	2.2%	13.3–16.7	33–34	12.8–16.7	32–34	29–46
Jiangwai	29.8	6.3%	12	4.5%	2.8	178.7	8.3%	72.2	6.4%	14.6–18.6	33–34	15.5–18.8	34–35	35–82
Zhoushan	43.8	9.2%	29.6	11%	1.1	175.1	8.1%	118.5	10.4%	16.9–22.1	30–34	16.9–22.1	34–35	33–64
Yushan	209.3	44.0%	145.1	53.7%	1.4	627.8	29.2%	435.3	38.4%	18.3–21.5	31–34	19.2–21.6	31–34	29–54

Wentai	158.1	33.2%	64.7	23.9%	2.7	790.7	36.8%	323.3	28.5%	16.7–21.8	29–35	17.2–21.8	29–34	20–81
Solenocera koelbeli														
November 2018 (Autumn)														
Zhouwai	76.4	3.2%	50.5	7.4%	2.2	305.5	2.2%	201.8	5.1%	21.2–22.6	33–34	21.2–22.6	33–34	60–73
Yushan	1792.8	75%	511.6	74.8%	3.6	10756.6	77.2%	3069.5	77.4%	21.7–22.5	33–34	19.6–22	34–35	65–84
Wentai	193.8	8.1%	51.8	7.6%	3.9	1550	11.1%	414	10.4%	21.7–23.9	32–35	18.5–21.6	34–35	51–100
Mindong	328.3	13.7%	69.9	10.2%	3.8	1313.1	9.4%	279.6	7.1%	22.9–23.7	33–35	17.5–20.9	34–35	76–104
January 2019 (Winter)														
Jiangwai	4.7	0.2%	5.9	1.5%	0.9	9.5	0.1%	11.9	0.4%	14.9–16	33–34	15.3–16.1	33–34	57–63
Zhoushan	38.3	1.5%	9	2.2%	4.3	153.2	0.8%	36	1.3%	15.4–16.6	33–34	15.5–16.7	33–35	55–66
Zhouwai	66.9	2.7%	14	3.5%	5.3	200.8	1.1%	42	1.5%	16.3–19.2	33–35	16–19.2	33–35	66–100
Yushan	1311.4	53.1%	184.5	45.7%	10.3	14425.3	77.1%	2029.6	72.1%	15–17.2	33–35	15.2–17.4	33–35	60–92
Wentai	904.6	36.6%	158	39.2%	4.8	3618.4	19.4%	632	22.4%	16.1–18.4	34–35	16.3–18.5	34–35	46–88
Mindong	145.6	5.9%	32	7.9%	4.6	291.2	1.6%	64	2.3%	17.6–19.6	34–35	18.3–19.4	34–35	50–100
May 2019 (Spring)														
Yushan	134.8	34%	16.8	34.1%	7.3	674	56.2%	83.9	56.4%	17.2–22	30–34	15.4–20.2	33–35	54–84
Mindong	262.2	66.1%	32.5	66.0%	7.1	524.5	43.8%	64.9	43.6%	24.6–24.8	34–35	18.6–22.8	34–35	75–98
August 2019 (Summer)														
Yushan	633.4	55.6%	121.7	62.4%	5.8	3166.9	55.6%	608.4	62.4%	27.7–28.9	31–34	21.3–28	33–35	69–84
Wentai	506.5	44.4%	73.3	37.6%	6.7	2532.4	44.4%	366.6	37.6%	26–28.7	33–34	17.3–26.9	33–35	75–97
May 2020 (Spring)														
Yushan	741.5	60.5%	168	77.4%	6.1	2224.4	53.5%	504.0	72.0%	19.4–20.2	31–33	17.2–20.8	34–35	39–68
Wentai	483.7	39.5%	48.9	22.5%	9.7	1934.8	46.5%	195.7	28.0%	22.5–25.3	31–34	18.6–23.7	34–35	33–49
August 2020 (Summer)														

Zhouwai	12.0	2.6%	1.5	1.5%	8.0	12.0	0.7%	1.5	0.4%	29.5	30	21.2	35	63.5
Yushan	456.3	97.4%	95.4	98.5%	7.1	1825.0	99.3%	381.7	99.6%	28.4–29.5	30–34	20.7–26	34–35	54–64
May 2021 (Spring)														
Wentai	38.1	100%	8.2	100%	5.6	152.5	100%	32.8	100%	20.6–23.6	29–34	18.1–23.1	30–35	24–81
November 2021 (Autumn)														
Zhouwai	479	2.9%	187.6	4.6%	2.2	3352.8	4.3%	1313.3	6.4%	18.6–25.7	30–34	18.6–22.5	32–34	52–76
Yushan	3871.2	23.6%	1174.8	28.7%	2.8	27098.2	34.5%	8223.4	40.2%	20.5–23.4	31–34	19.2–22.4	34–35	46–97
Wentai	12050.3	73.5%	2729.7	66.7%	4.3	48201.3	61.3%	10918.7	53.4%	21.7–24.2	32–34	18.8–24.7	34–35	53–77
June 2022 (Spring)														
Yushan	1321	100%	248	100%	6.5	2642	100%	496	100%	23.5–23.9	29–31	18.2–22.6	31–34	18–54
September 2022 (Summer)														
Yushan	297.7	100%	37.7	100%	8.7	893.1	100%	113	100%	25.4–26.6	34–35	19.5–22.1	34–35	62–91
December 2022 (Winter)														
Zhoushan	85	100%	212.4	100%	4.1	255.1	100%	637.3	100%	15–18.3	33–34	16–18.5	33–34	36–56
May 2023 (Spring)														
Yushan	124.4	100%	108.2	100%	1.9	248.8	100%	216.4	100%	19–20.3	34–35	18.2	34–35	70–83
August 2023 (Summer)														
Yushan	935.5	100%	141.5	100%	6.4	2806.6	100%	424.6	100%	29–29.5	34–35	21.8–24.7	34–35	51–66
November 2023 (Autumn)														
Zhoushan	176.6	10.1%	116.3	19.5%	1.5	353.1	7.4%	232.6	14.8%	18.3–22	34–35	21.1–22.1	34–35	54–68
Yushan	1241.6	70.8%	381.9	64.1%	3	3724.8	78.4%	1145.6	72.8%	22.6–23	30–34	17.9–18.3	34–35	60–93
Wentai	336	19.2%	97.8	16.4%	3.4	672	14.1%	195.6	12.4%	22.8–23.7	34–35	18.2–18.4	34–35	72–84
December 2023 (Winter)														
Zhoushan	256.2	12.1%	147.9	28.4%	5.8	1024.6	7.8%	591.6	19.6%	17.4–19	34–35	17.4–19	34–35	52–70

Yushan	1051.9	49.8%	200	38.5%	5.2	7363.2	55.7%	1399.7	46.3%	16.3–19.3	32–34	17.6–19.3	33–34	50–71
Wentai	804.7	38.1%	172.2	33.1%	4.7	4828.3	36.5%	1033.2	34.2%	14.7–19.8	31–35	17.5–19.6	33–34	40–89
April 2024 (Spring)														
Zhoushan	152.3	4.7%	28.3	5.5%	5.7	1370.6	13%	254.6	14.9%	13.4–17.7	28–34	13–15.1	33–34	49–83
Yushan	639.3	19.9%	104.4	20.3%	6.5	1917.9	18.2%	313.1	18.3%	16.8–18.1	30–33	17.5–18.8	34–35	44–67
Wentai	2414.9	75.3%	381	74.2%	4.3	7244.8	68.8%	1143.1	66.8%	22.8–24.1	34–35	18.3–20.9	34–35	62–85
December 2024 (Winter)														
Zhouwai	871.8	53.6%	175.3	53.3%	5.4	2615.3	34.3%	525.8	33.9%	21.2–23.4	34–35	19.7–23.4	34–35	60–88
Yushan	487.2	30%	102.1	31%	3.6	3410.7	44.7%	715	46.1%	15.8–24.6	31–34	16.2–20.7	31–34	24–111
Wentai	266.1	16.4%	51.6	15.7%	5.6	1596.3	20.9%	309.7	20%	16.5–18.2	32–34	16.8–19.3	33–34	33–93
April 2025 (Spring)														
Yushan	357.3	11.7%	76.8	19.1%	4.8	1786.4	14.3%	384	22.8%	16.3–23.3	32–34	15.7–19.2	34–35	61–81
Wentai	2686.2	88.3%	324.3	80.8%	8.8	10744.8	85.7%	1297	77.2%	22.5–24.8	34–35	17.6–22.2	34–35	51–100
August 2025 (Summer)														
Zhouwai	1276.5	78.7%	329.7	77.3%	4.4	3829.4	61.3%	989	59.4%	29.4–30.8	33–34	20.7–22.4	34–35	66–85
Yushan	346	21.3%	96.7	22.7%	4.6	2421.7	38.7%	677	40.6%	29–30.5	33–34	18.7–22.8	34–35	50–101
November 2025 (Autumn)														
Zhouwai	34.4	4%	15.9	6.1%	2.1	103.1	3.2%	47.7	4.7%	16.9–22	21–34	16.9–22.5	34–35	54–82
Yushan	122.4	14.4%	14.7	5.6%	5.7	367.2	11.3%	44	4.3%	22.4–23.1	34–35	19.7–21.9	34–35	59–100
Yuwai	695.8	81.6%	231.7	88.3%	4.2	2783.2	85.5%	927	91%	18.3–21.8	31–35	18.4–21.8	34–35	41–67

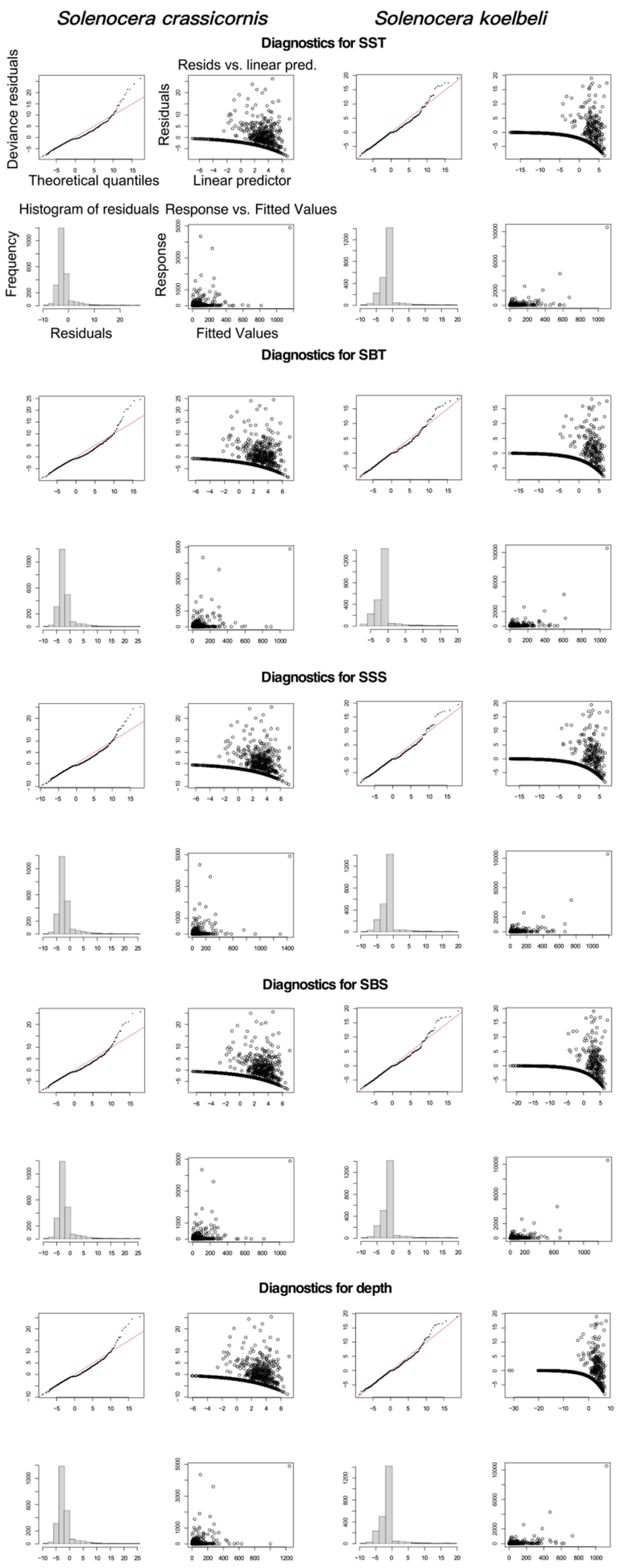
Solenocera crassicornis
Solenocera koelbeli
Diagnostics for SST
Resids vs. linear pred.
Deviance residuals
Theoretical quantiles
Residuals
Linear predictor
Histogram of residuals
Response vs. Fitted Values
Frequency
Response
Residuals
Fitted Values
Diagnostics for SBT
Diagnostics for SSS
Diagnostics for SBS
Diagnostics for depth

Figure S1. Residual diagnostic plots for the generalized additive model of environmental variables such as sea surface temperature, sea surface salinity, sea bottom temperature, sea bottom salinity and depth for shrimp species *Solenocera crassicornis* and *Solenocera koelbeli*. Each group contains four subgraphs: deviance residuals against linear predictors, Q-Q normal quantile plots, response versus fitted values, and histograms of residuals.

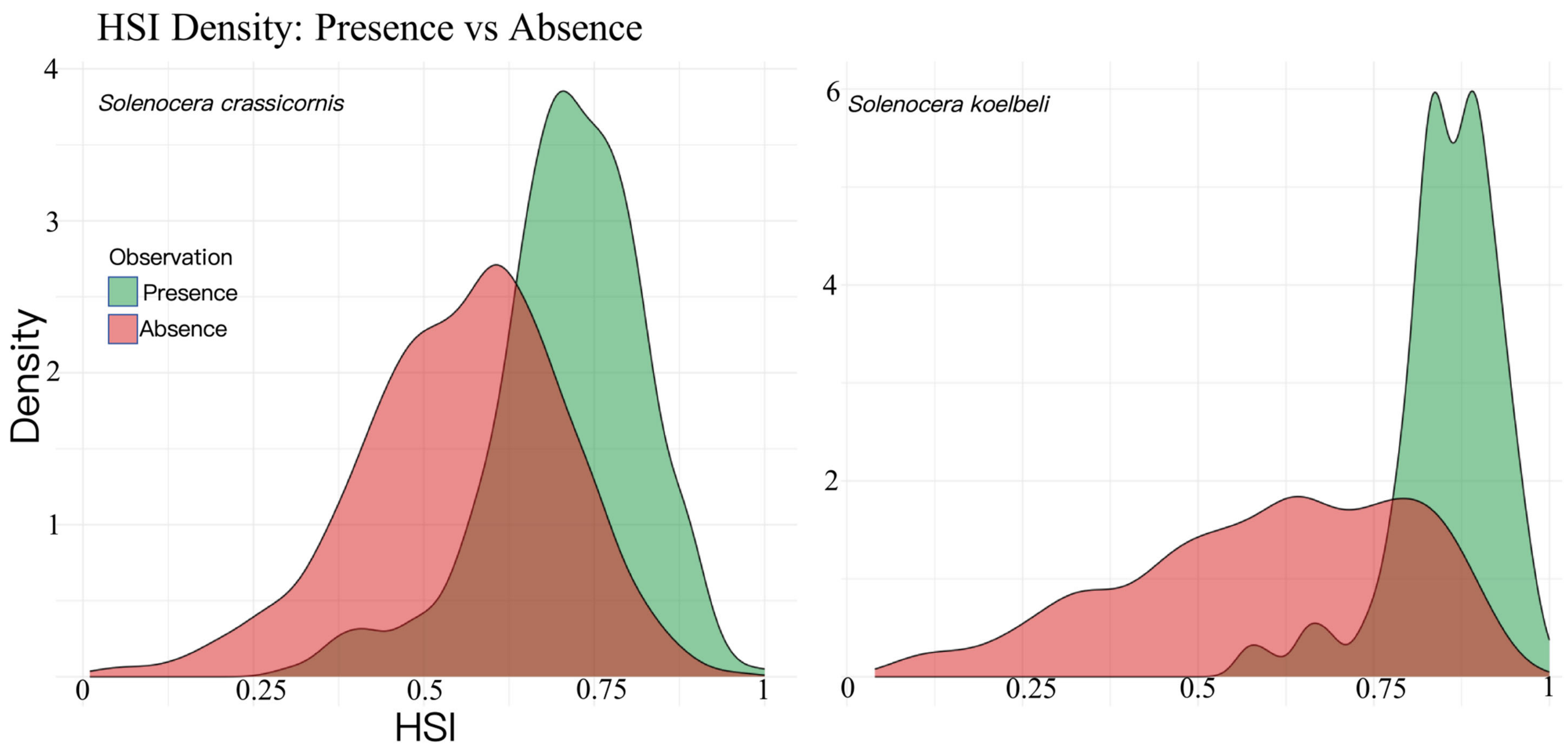

Figure S2. Habitat suitability index (HSI) density distributions for two shrimp species: *Solenocera crassicornis* and *Solenocera koelbeli*. Density curves are separated by observation status (presence vs. absence) to illustrate differences in HSI value frequency between sampling sites with and without target shrimp occurrence.

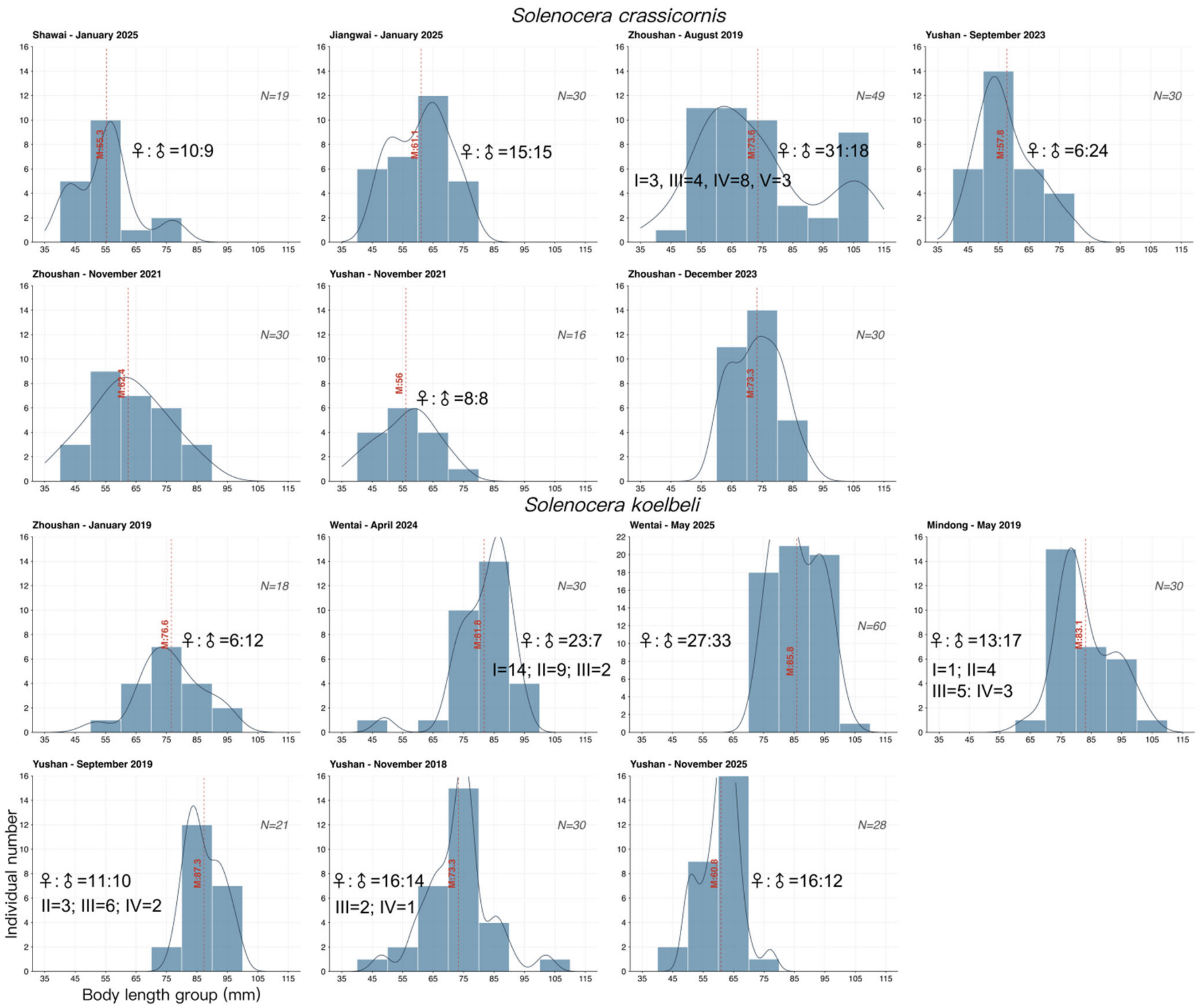

Solenocera crassicornis
Shawai - January 2025
N=19
♀:♂=10:9
Jiangwai - January 2025
N=30
M:61.1
♀:♂=15:15
Zhoushan - August 2019
N=49
M:73.6
♀:♂=31:18
I=3, III=4, IV=8, V=3
Yushan - September 2023
N=30
M:57.8
♀:♂=6:24
Zhoushan - November 2021
N=30
M:62.4
Yushan - November 2021
N=16
M:56
♀:♂=8:8
Zhoushan - December 2023
N=30
M:73.3
Solenocera koelbeli
Zhoushan - January 2019
N=18
M:76.6
♀:♂=6:12
Wentai - April 2024
N=30
M:81.8
♀:♂=23:7
I=14; II=9; III=2
Wentai - May 2025
N=60
M:85.8
♀:♂=27:33
Mindong - May 2019
N=30
M:83.1
♀:♂=13:17
I=1; II=4
III=5: IV=3
Yushan - September 2019
N=21
M:87.3
♀:♂=11:10
II=3; III=6; IV=2
Yushan - November 2018
N=30
M:73.3
♀:♂=16:14
III=2; IV=1
Yushan - November 2025
N=28
M:60.8
♀:♂=16:12
Individual number
Body length group (mm)

Figure S3. Length-frequency distributions of *Solenocera crassicornis* and *Solenocera koelbeli* from different sampling sites, months and years. Notes: Red dashed lines indicate mean body length; *N* is sample size; ♀:♂represents female-to-male sex ratio; roman numerals (I-V) denote gonadal development stages.

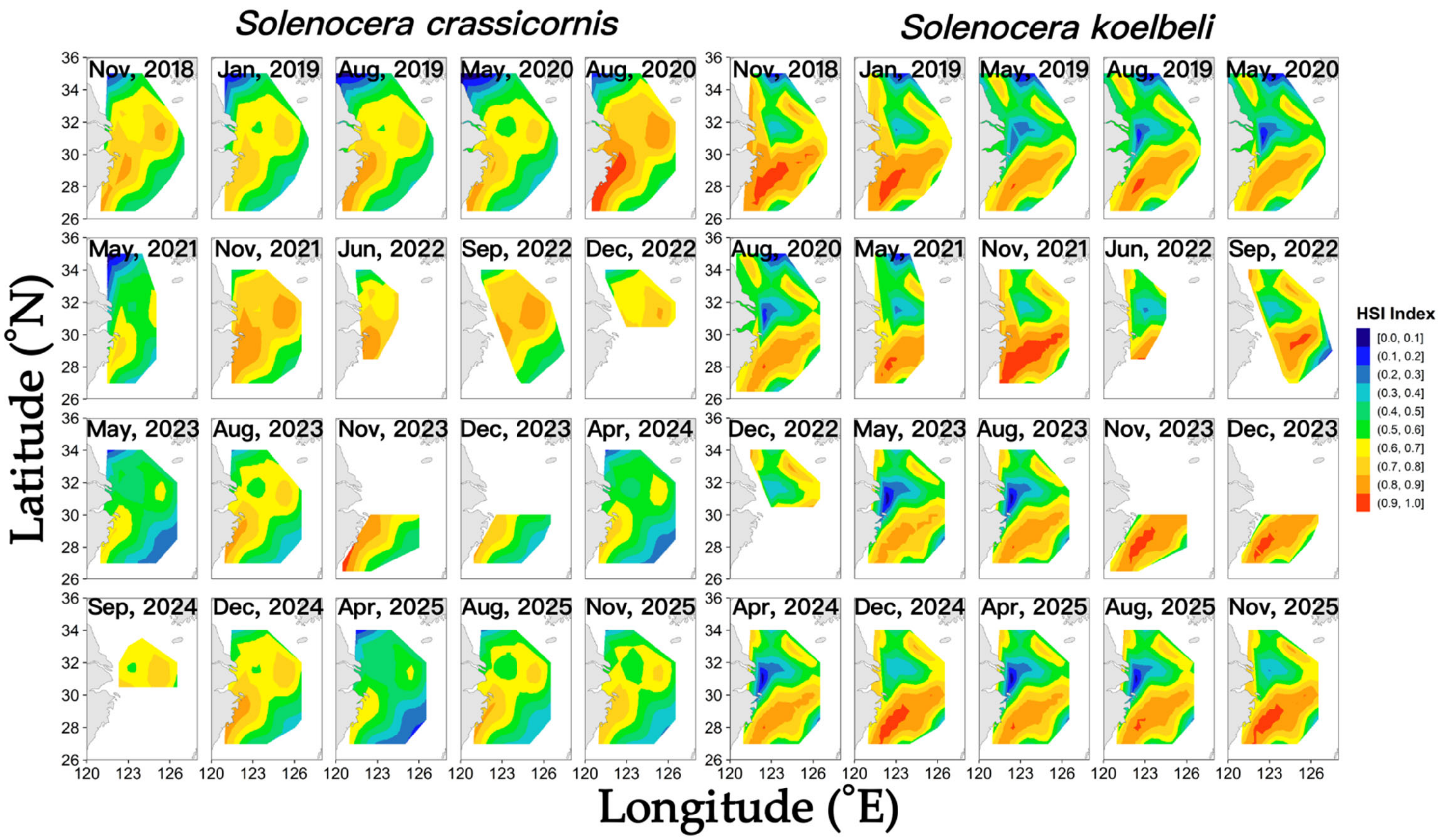

Figure S4. Spatiotemporal variations in habitat suitability index (HSI) for *Solenocera crassicornis* (left panels) and *S. koelbeli* (right panels) across the study area from November 2018 to November 2025. Colour gradients represent HSI values ranging from 0 to 1; higher values indicate more suitable habitats for the two

shrimp species. No catch of *S. crassicornis* was recorded in May 2019, and no catch of *S. koelbeli* in September 2024. Thus, data from these two months are excluded from the results.
